# Lhx6 regulates canonical Wnt signaling to control the fate of mesenchymal progenitor cells during mouse molar root patterning

**DOI:** 10.1371/journal.pgen.1009320

**Published:** 2021-02-17

**Authors:** Jinzhi He, Junjun Jing, Jifan Feng, Xia Han, Yuan Yuan, Tingwei Guo, Fei Pei, Yuanyuan Ma, Courtney Cho, Thach-Vu Ho, Yang Chai

**Affiliations:** 1 Center for Craniofacial Molecular Biology, Herman Ostrow School of Dentistry, University of Southern California, Los Angeles, California, United States of America; 2 State Key Laboratory of Oral Diseases, National Clinical Research Center for Oral Diseases, West China Hospital of Stomatology, Chengdu, Sichuan province, China; Children’s Hospital of Philadelphia, UNITED STATES

## Abstract

Mammalian tooth crown formation has long served as a model for investigating how patterning and morphogenesis are orchestrated during development. However, the mechanism underlying root patterning and morphogenesis remains poorly understood. In this study, we find that Lhx6 labels a subpopulation of root progenitor cells in the apical dental mesenchyme, which is closely associated with furcation development. Loss of Lhx6 leads to furcation and root number defects, indicating that Lhx6 is a key root patterning regulator. Among the multiple cellular events regulated by Lhx6 is the odontoblast fate commitment of progenitor cells, which it controls in a cell-autonomous manner. Specifically, *Lhx6* loss leads to elevated expression of the Wnt antagonist *Sfrp2* and down-regulation of Wnt signaling in the furcation region, while overactivation of Wnt signaling in Lhx6+ progenitor cells partially restore the furcation defects in *Lhx6*^*-/-*^ mice. Collectively, our findings have important implications for understanding organ morphogenesis and future strategies for tooth root regeneration.

## Author summary

In the mammalian dentition, the number of the tooth roots varies in a manner that is finely tailored to the physiological function of each tooth type. Specifically, the anterior incisors and canines, which are mainly responsible for lower occlusal pressure movements such as cutting or shearing, are single-rooted, while the more posterior premolars and molars are multi-rooted and used for motions that require higher bite force such as chewing, crushing and grinding. So far, we have very limited understanding of the molecular mechanism that precisely determines the number of tooth roots. The multi-rooted mouse molar presents an excellent model for us to study this question. Here, we show that the gene *Lhx6* is a key determinant of mouse molar root number, as the absence of this gene results in the transformation of multi-rooted mouse molars into single-rooted ones. We further show how this gene achieves its functional specificity in determining the tooth root number by controlling the region-specific fate determination of progenitor cells. This finding not only provides clues that could inform future tooth regeneration strategies, but also serves to illustrate developmental processes shared with other mammalian organs.

## Introduction

Teeth are biomineralized organs in the oral cavity that support physiological functions including eating, pronunciation, and facial esthetics. Each tooth can be divided into two parts, the crown and the root. The root is the lower two-thirds of the tooth embedded in the jawbone to which it is anchored. Morphological features of roots, including both their length and number, vary across the mammalian dentition. Investigating how root morphology is patterned is of significance for several reasons. Uncovering the mechanisms by which root morphology is regulated provides clues that may reveal common principles of developmental biology [1]. A comprehensive understanding of how root morphology is determined also serves as a prerequisite for tooth root regeneration, because root shape and number are tightly connected to the unique physiological function of each particular type of tooth. In addition, tooth root traits provide crucial evidence that elucidates the evolutionary history of hominins [2–4]. However, the molecular mechanism that regulates tooth root patterning and morphogenesis remains largely unclear [5].

Recent studies have identified signaling networks that determine root length, including BMP, Wnt, SHH, TGF-β and PTHrP–PPR [6–14]. However, we still do not have a clear understanding of how teeth with multiple roots are patterned and how the specific number of roots is determined [15–18]. One of the crucial determinants of the tooth root number is the development of the furcation, a structure that separates the roots of mammalian posterior teeth. Previous studies suggested that furcation development might be controlled by regulatory clues from either Hertwig’s epithelial root sheath (HERS) or the cranial neural crest-derived dental mesenchyme (for review, see [5]). We have recently highlighted the pivotal role of the dental mesenchyme in regulating furcation development and tooth root patterning [1]. Specifically, deletion of Ezh2 in the dental mesenchyme, but not in the dental epithelium, results in the transformation of maxillary and mandibular molars from multi-rooted to single-rooted [1]. Since Ezh2 is broadly expressed throughout the dental mesenchyme [1], the identity of the specific subpopulation of dental mesenchymal cells associated with furcation formation, as well as the underlying mechanisms, are yet to be elucidated.

LIM-homeobox genes are a large family encoding LIM-homeodomain transcriptional factors that include two LIM domains for interacting with other proteins and a DNA-binding homeodomain for interacting with target genes [19,20]. Members of this family play crucial roles in mediating tissue patterning [19]. For example, Tzchori et al. have shown that *Lhx2*, *Lhx9* and *Lmx1b* work together to control mouse limb patterning and growth [21]. Another LIM-homeobox gene, *Lhx6*, is expressed during mouse embryogenesis in the basal forebrain and domains of the first pharyngeal arch [22]. In the brain, Lhx6 is an important regulator specifying cortical interneuron fate [23,24]. During craniofacial development, *Lhx6* is a key patterning gene, as its expression pattern defines the odontogenic domain within the mandibular and maxillary processes where odontogenesis occurs [22,25,26]. During embryonic tooth crown development, *Lhx6* is restricted to the cranial neural crest-derived dental mesenchyme, where it has a high expression level [22,27]. However, loss of Lhx6 does not lead to crown malformation in mouse molars [27].

In this study, we show *in vivo* evidence that Lhx6 is a key regulator of tooth root patterning. *Lhx6* is consistently and specifically expressed in the dental mesenchyme in mice after birth. Unexpectedly, despite the fact that *Lhx6* is broadly expressed in dental mesenchyme during embryonic tooth crown development, it labels a subpopulation of Gli1+ root progenitor cells in the apical dental mesenchyme during postnatal root development. A subpopulation of the progeny of Lhx6+ cells are closely associated with root furcation development, and loss of Lhx6 results in root furcation and number defects. Mechanistically, Lhx6 regulates tooth root patterning and morphogenesis through coordinating key cellular events including mesenchymal proliferation and differentiation as well as dental epithelial elongation. Specially, Lhx6 controls furcation development through regulating the odontoblast fate commitment of progenitor cells by inhibiting canonical Wnt signaling activity. Our study reveals how mesenchymal Lhx6 achieves its functional specificity in regulating tooth root patterning, and highlights the importance of dental mesenchymal regulation for root patterning and morphogenesis.

## Results

### Molar furcation and root elongation regions show distinct cellular dynamics

**Fig 1:**
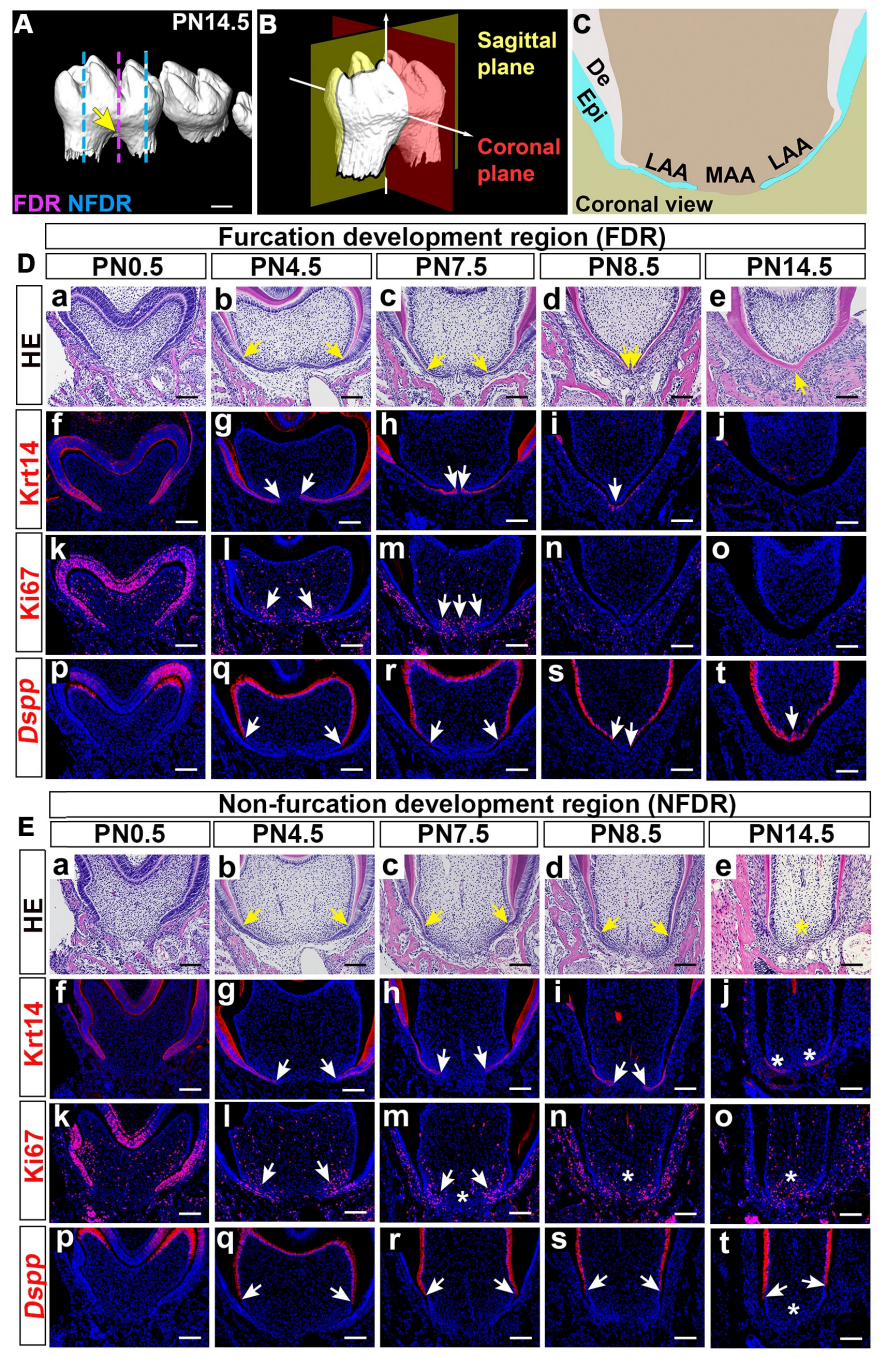
Postnatal furcation development of the mandibular first molar. (A) MicroCT scanning of mandibular first molars from control mice at PN14.5 showing the furcation morphology. Arrow indicates tooth root furcation. The pink line indicates furcation development region (FDR); blue lines indicate non-furcation development region (NFDR). (B) Schematic drawing of the coronal and sagittal planes of mouse molars. (C) Schematic drawing of the lateral apical area (LAA) and middle apical area (MAA) in coronal sections of the developing tooth root. (D) H&E staining (a-e), immunofluorescence staining of epithelial marker Krt14 (f-j) and cell proliferation marker Ki67 (k-o), and *in situ* RNAscope of odontoblast differentiation marker *Dspp* (p-t) in the FDR of mandibular first molars in the coronal view from control mice at indicated stages. (E) H&E staining (a-e), immunofluorescence staining of Krt14 (f-j) and Ki67(k-o), and RNAscope *in situ* hybridization of *Dspp* (p-t) of the NFDR of mandibular first molars in the coronal view from control mice at indicated stages. Arrows in b-e of D, E indicate the ends of dentin protrusions elongating from the buccal and lingual sides; in g-i of D, E indicate the tips of the dental epithelial processes; in l-m of D, E indicate regions with actively mesenchymal cell proliferation activity; and in q-t of D and E indicate the most apically located cells expressing *Dspp*. Asterisks in E indicate the differences present in the NFDR in comparison to the FDR. Scale bars: 100μm in A; 200μm in D and E. Epi, epithelium; De, dentin.

The furcation is the structure at the bottom of the dental pulp chamber that separates the roots of multi-rooted teeth (Fig 1A). Thus, studying furcation development is crucial for understanding tooth root patterning, as the furcation is intimately connected to the determination of the root number. To investigate the furcation development process, we systematically analyzed coronal sections (as defined in Fig 1B) of the mandibular first molars of control mice from PN0.5 to PN14.5, during which the furcation forms. We divided the developing tooth root into two parts: the furcation development region (FDR) in the middle and the non-furcation development region (NFDR) where root elongation occurs on either side (Fig 1A). Within each of these two parts, we further divided the apical portion into the lateral apical area (LAA) and middle apical area (MAA) (Fig 1C) for downstream analysis.

We compared cellular events between the FDR (Fig 1D) and NFDR (Fig 1E), including epithelial elongation, mesenchymal cell proliferation and differentiation. We found these two regions started to show differences after PN4.5. In the FDR, HERS elongated horizontally, formed a bridge across the LAA and MAA at PN8.5 (Fig 1D/f-i/), then lost its bilayered structure completely (Fig 1D/j/). In the NFDR, HERS did not fuse in the MAA, and the tip of HERS maintained its bilayered structure for a longer period (Fig 1E/f-j/). The proliferating dental mesenchymal cells in the FDR expanded from the LAA to the MAA (Fig 1D/k-m/), and the mesenchymal cell proliferation diminished and eventually disappeared in both the LAA and the MAA after PN7.5 (Fig 1D/n-o/). However, there was a difference in the dynamics of cell proliferation in the FDR and NFDR, as mesenchymal cells in the NFDR kept actively proliferating after PN7.5 (Fig 1E/k-o/). In addition, mesenchymal cells in the LAA and MAA differentiated into odontoblasts sequentially (lateral to medial) in the FDR (Fig 1D/p-t/), enabling the dentin to elongate apically and form the hard tissue bridge in the middle. In contrast, in the NFDR, only the mesenchymal cells in the LAA underwent odontoblast differentiation (Fig 1E/p-t/). Therefore, the dentin in the NFDR kept elongating apically without fusion in the MAA. To summarize our results, the cellular dynamics of FDR and NFDR are differently patterned, which may contribute to the morphological difference between furcation development and root elongation. The distinctly patterned cellular dynamics in the FDR and NFDR of the developing tooth root suggest that there may be a specific group of genes/molecules which control the location-specific fate of dental cells and participate in furcation development and root patterning.

### Lhx6+ cells are a subpopulation of Gli1+ progenitor cells closely associated with furcation development

**Fig 2:**
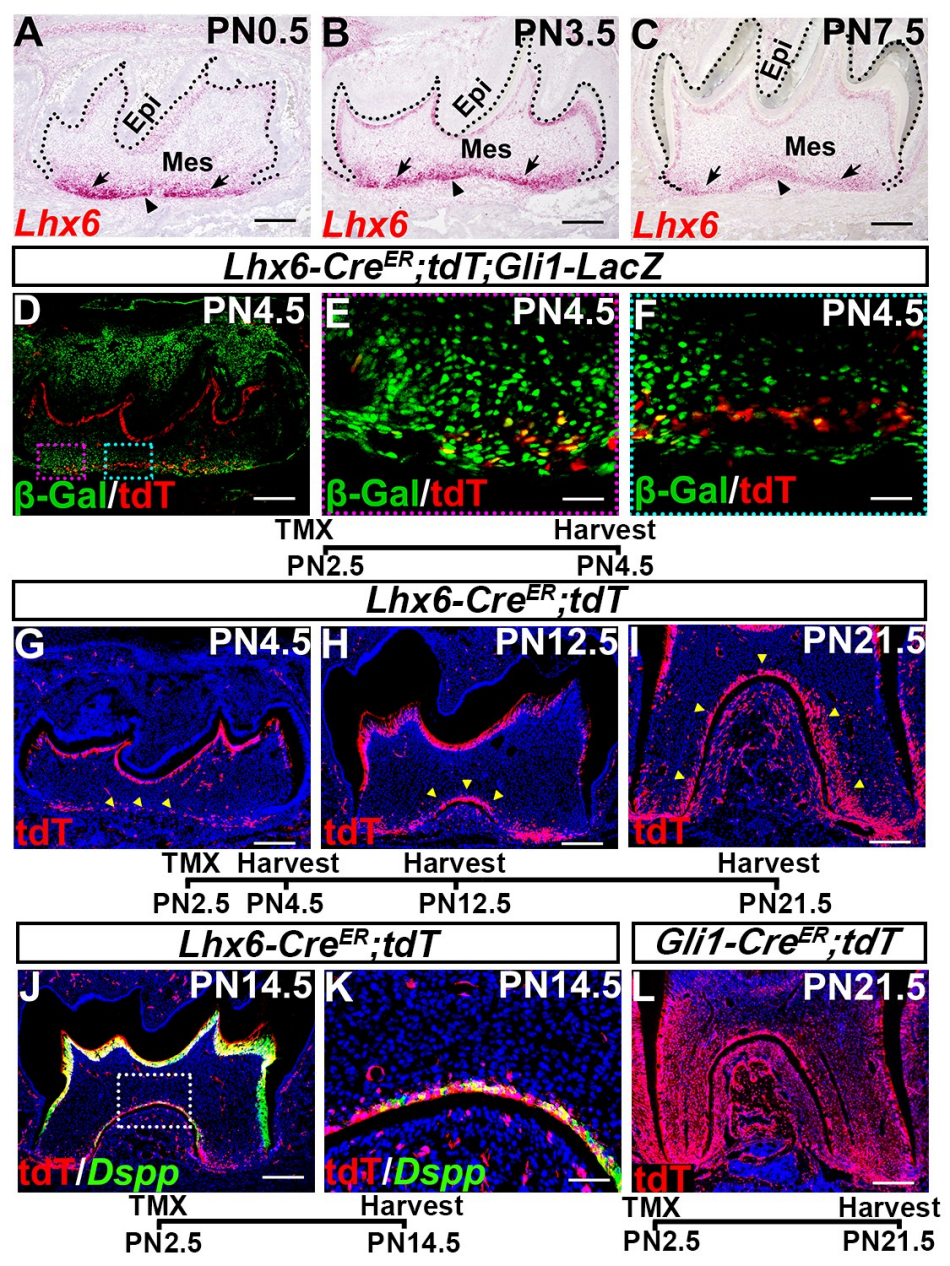
Lhx6 labels a subpopulation of Gli1+ progenitor cells closely associated with furcation development. (A-C) RNAscope assays at indicated stages showing expression pattern of *Lhx6* during postnatal root morphogenesis. Sagittal sections of mandibular first molars were analyzed. Dotted lines indicate border between dental epithelium and mesenchyme. Epi, epithelium; Mes, mesenchyme. Arrows indicate non-furcation development region (NFDR), and arrowheads point to furcation development region (FDR). (D-F) Co-localization of tdTomato (as indicated by red signal) with β-gal (as indicated by green signal) within mandibular first molars of PN4.5 *Lhx6-Cre*^*ER*^*;tdT;Gli1-LacZ* mice after tamoxifen injection at PN2.5. Sagittal sections of mandibular first molars were analyzed. Yellow signal indicates cells expressing both Lhx6 and Gli1. Red box in D is shown magnified in E, and blue box in D is shown magnified in F. (G-I) Lineage tracing of Lhx6*+* cells in mandibular first molars of PN4.5, PN12.5 and PN21.5 *Lhx6-Cre*^*ER*^*;tdT* mice after tamoxifen injection at PN2.5. Sagittal sections of mandibular first molars were analyzed. Arrowheads indicate Lhx6+ cells and their progeny. (J-K) Progeny of Lhx6+ cells (as indicated by red signal) in the furcation express odontoblast differentiation marker *Dspp* (as indicated by green signal) in mandibular first molars of PN14.5 *Lhx6-Cre*^*ER*^*;tdT* mice after tamoxifen injection at PN2.5. Sagittal sections were analyzed. Box in J is shown magnified in K. (L) Lineage tracing of Gli1*+* cells in mandibular first molars of PN21.5 *Gli1-Cre*^*ER*^*;tdT* mice after tamoxifen injection at PN2.5. Sagittal sections of mandibular first molars were analyzed. The schematic at the bottom of each set of panels indicates the induction protocol. Scale bars: 200μm in A, B, C, D, G, H, I, J, L; 50μm in K; 20μm in E, F. TMX: tamoxifen.

*Lhx6* is a key patterning gene during early craniofacial development [25,26]. Since genes that are important for embryonic development usually have a role in the postnatal growth of the same tissue, it is likely that *Lhx6* may be involved with postnatal root patterning. To specifically investigate the role of *Lhx6* in tooth development, we first analyzed its expression pattern from E13.5 to PN7.5. We found that *Lhx6* was specifically expressed in the dental mesenchymal cells at both prenatal and postnatal stages of tooth development (Figs 2A–2C and S1). Consistent with previous findings [22,27], at the bud and cap stages, *Lhx6* was ubiquitously expressed in the dental mesenchyme (S1A and S1B Fig). However, from E16.5 onwards, *Lhx6* expression was enriched in the apical region (S1C Fig). This enrichment of *Lhx6* transcripts in the apical dental mesenchyme persisted after birth, although its overall expression level decreased gradually (Fig 2A–2C). This apical enrichment of *Lhx6* expression prior to tooth root development suggested that it might play a crucial role in tooth root patterning and morphogenesis.

Gli1+ cells supporting mouse molar root growth also reside in the apical region of dental mesenchyme [7,11]. The finding that Lhx6+ cells populate the same apical region as the Gli1*+* cells suggested that these two cell populations are closely related. To elucidate this relationship, we generated *Lhx6-Cre*^*ER*^*;tdT;Gli1-LacZ* reporter mice, in which the cells expressing tdTomato and beta-galactose represent Lhx6+ and Gli1+ cells, respectively. Tamoxifen was injected at PN2.5, and the mandibles were collected two days after induction. We noticed that Tdtomato signal could be detected from both the odontoblasts of the crown and apical dental mesenchymal cells (Fig 2D), which is consistent with the *in situ* RNAscope results (Fig 2A–2C). As shown in Fig 2D–2F, Lhx6+ cells co-localized with some Gli1*+* cells in the apical dental mesenchyme, suggesting that these apical Lhx6+ cells are a subpopulation of Gli1*+* cells.

Considering that Gli1*+* cells are a heterogeneous population containing tooth root progenitor cells that contribute to the entire dental mesenchyme [7,11], we further tested the contribution of Lhx6+ cells to tooth root development. We found that progeny of labeled Lhx6+ cells were closely associated with furcation development during root development, as strong tdTomato signal was detected predominantly in the furcation region compared to other parts of the tooth root mesenchyme (Fig 2G–2I). We confirmed that some cells derived from the *Lhx6+* lineage (tdTomato+ cells) overlapped with expression of odontoblast marker *Dspp*, indicating Lhx6+ cells can differentiate into odontoblasts in the furcation (Fig 2J–2K). In comparison to Gli1*+* progeny, the Lhx6*+* cells only contributed to a subset of the root dental mesenchyme (Fig 2I and 2L), which supported our observation that *Lhx6* labels a subpopulation of Gli1*+* cell population during root development. To summarize our findings, Lhx6+ cells are a subpopulation of Gli1*+* root progenitor cells, and these cells are closely associated with furcation formation during tooth root development.

### Loss of Lhx6 leads to furcation and root patterning defects in mouse molars

Combining the expression pattern of *Lhx6* with lineage tracing results led us to hypothesize that Lhx6 may serve as a key regulator for tooth furcation development and root patterning. To test this hypothesis, we generated *Lhx6* knockout mice using the *Lhx6-Cre*^*ER*^ mouse line. *The Lhx6-Cre*^*ER*^ knock-in allele in these mice abolishes *Lhx6* gene function; therefore, homozygous mice (referred to here as *Lhx6*^*-/-*^ unless otherwise specified) have a knockout phenotype similar to other null mutations of this gene [28]. These *Lhx6*^*-/-*^ mice were born alive and appeared grossly normal but later showed an obvious reduction in body size compared to controls (S2A and S2B Fig). The *Lhx6*^*-/-*^ mice ultimately died three to four weeks after birth; however, we were still able to observe their molar development because by PN18.5 tooth root development is complete and the molars have erupted into the oral cavity [5,11].

**Fig 3:**
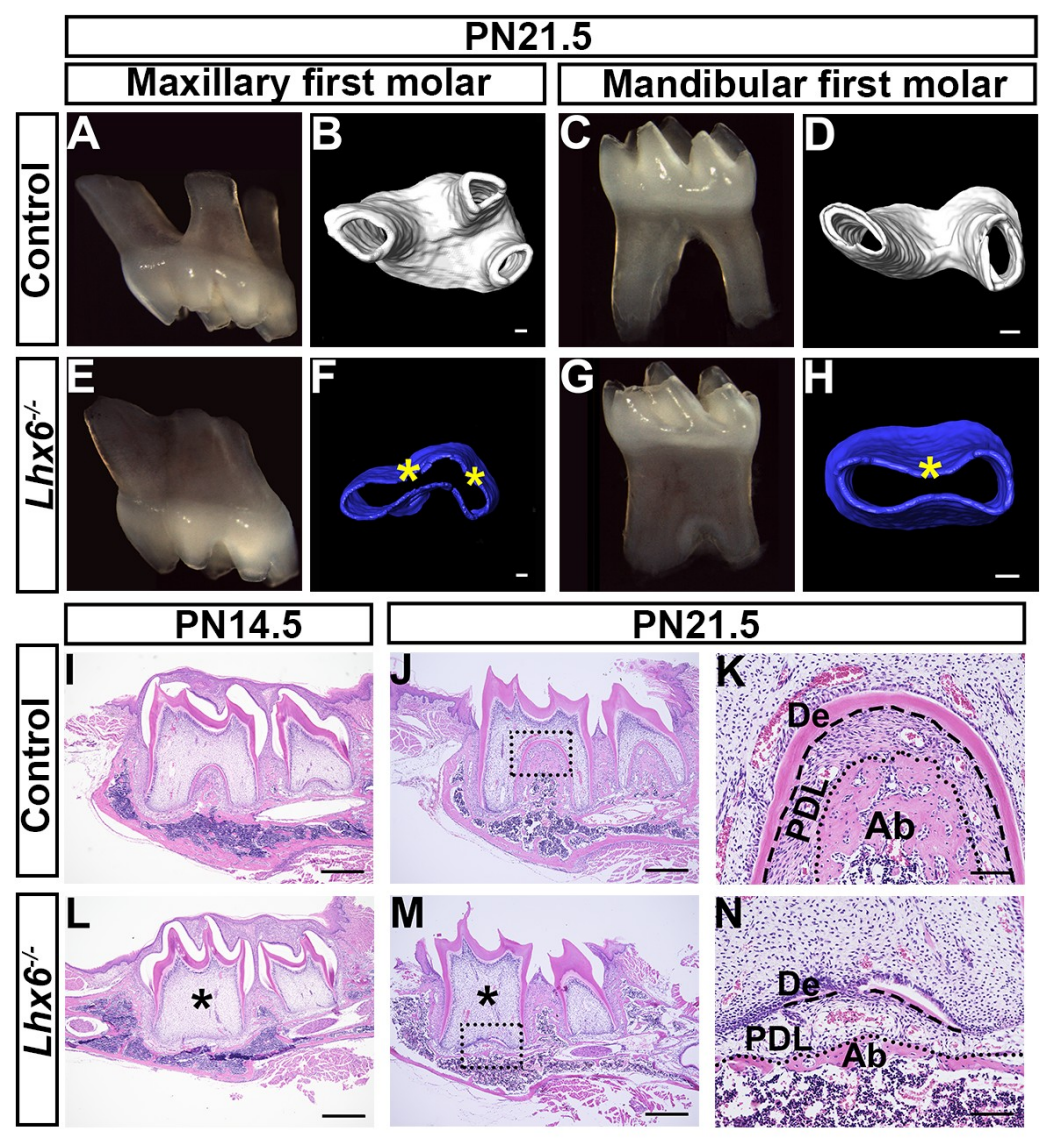
Loss of *Lhx6* leads to furcation defects in mouse molars. (A-B) Image and microCT scanning of maxillary first molars of PN21.5 control mice. (C-D) Image and microCT scanning of mandibular first molars of PN21.5 control mice. (E-F) Image and microCT scanning of maxillary first molars of PN21.5 *Lhx6*^*-/-*^ mice. (G-H) Image and microCT scanning of mandibular first molars of PN21.5 *Lhx6*^*-/-*^ mice. (I-N) H&E staining of mandibular first molars of control (I-K) and *Lhx6*^*-/-*^ mice (L-N) at indicated stages in sagittal view. K and N are magnified images of the boxes in J and M, respectively. Asterisks in F, H, L and M indicate absence of furcation. Dashed lines in K and N indicate the border between tooth hard tissues and periodontal ligament, while dotted lines in K and N indicate the border between periodontal ligament and alveolar bone. Scale bars: 500μm in I, J, L, M; 100μm in D and H; 90μm in B and F. De, dentin; PDL, periodontal ligament; Ab, alveolar bone.

We first confirmed the Lhx6 protein was ablated in the molar mesenchyme of *Lhx6*^*-/-*^ mice at the newborn stage (S2C and S2D Fig). We analyzed the phenotypes of both maxillary and mandibular first molar roots from PN4.5 to PN21.5. We found that the tooth crowns were normal at PN4.5, when crown formation completes (S2E and S2F Fig), and continued appearing normal through PN21.5 (Fig 3). The lengths of the tooth roots in *Lhx6*^*-/-*^ mice were mildly affected (Fig 3A–3H). Strikingly, both the maxillary and mandibular first molars failed to form a furcation, leading to a change in their number of roots. In control mice there were three roots in the maxillary first molar (Fig 3A and 3B) and two in the mandibular first molar (Fig 3C and 3D). However, both the maxillary (Fig 3E and 3F) and mandibular first molars (Fig 3G and 3H) were single-rooted in *Lhx6*^*-/-*^ mice. To investigate the mechanisms underlying the tooth root phenotype, we used the mandibular first molar as a model for further studies. Histological analysis further confirmed microCT scanning results (Fig 3I–3N). In *Lhx6*^*-/-*^ mice, dentin could not be detected in the furcation region at PN14.5 (Fig 3L), and the furcation failed to form even at PN21.5 (Fig 3M). Furthermore, periodontal tissues in the furcation region of the *Lhx6*^*-/-*^ mice were also abnormal: the alveolar bone was underdeveloped, and periodontal ligament development was also impaired (Fig 3K and 3N).

We sought to investigate why loss of Lhx6 leads to tooth root malformation without disturbing crown development, although it is expressed both during crown and root development. Previous studies have shown that there is functional redundancy between Lhx6 and Lhx8 in regulating early tooth development, as single mutation of either *Lhx6* or *Lhx8* does not affect crown development, while double mutations result in an absence of tooth germ formation [27,29,30]. We found that the expression patterns of *Lhx6* and *Lhx8* largely overlap during crown development, suggesting that one of these genes could compensate for the loss of the other during this process (S3A–S3D Fig). However, at E16.5 when crown patterning has almost completed but root patterning has not yet started, the expression patterns of *Lhx6* and *Lhx8* started to differ (S3E–S3H Fig). During the postnatal root development stage, *Lhx8* was highly expressed in pre-odontoblasts but not in the apical region where *Lhx6* transcripts were highly enriched (S3I–S3L Fig). This dynamic *Lhx6/8* co-localization pattern may partly explain why loss of Lhx6 produced an abnormal tooth root phenotype without affecting the crown.

### Lhx6 regulates mesenchymal proliferation and epithelial elongation during tooth root patterning and morphogenesis

**Fig 4:**
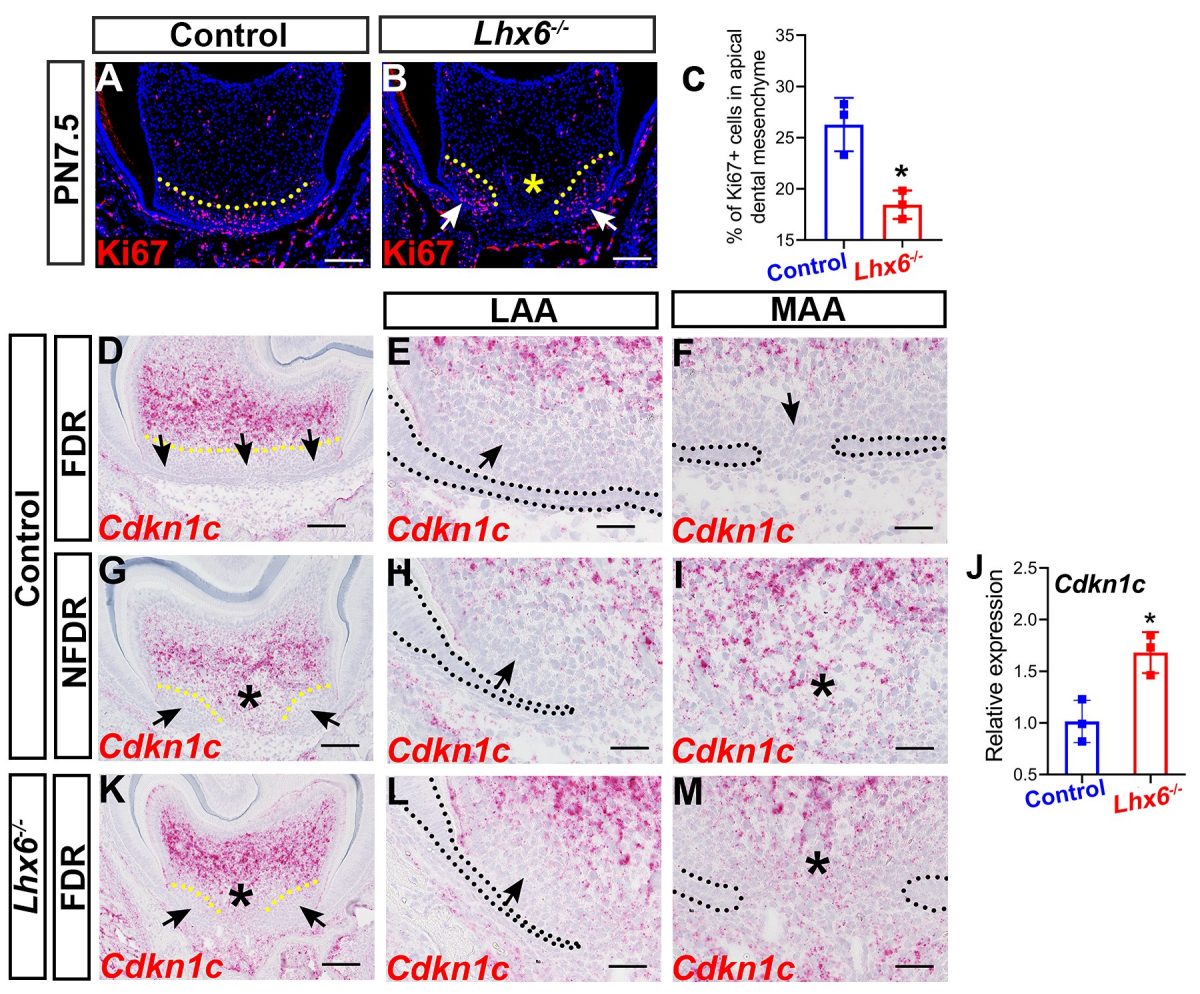
Loss of *Lhx6* compromises dental mesenchymal proliferation in the furcation development region. (A-B) Ki67 staining of FDR from control and *Lhx6*^*-/-*^ mice at PN7.5. Coronal sections of mandibular first molars were analyzed. Yellow dotted lines indicate the border of the actively proliferating apical mesenchymal region. Arrows in B indicate the region where proliferating cells reside in the *Lhx6*^*-/-*^ mice, and asterisk in B indicates significantly decreased cell proliferation activity in the MAA of *Lhx6*^*-/-*^ mice. (C) Comparison of Ki67+ mesenchymal cells in the apical regions of FDR between control and *Lhx6*^*-/-*^ mice at PN7.5. The data represented as mean ± SD. The asterisk indicates p < 0.05. (D-I) Distribution pattern of *Cdkn1c* in control mice at PN4.5. Coronal sections of mandibular first molars were analyzed. E and H are magnified images of the LAA in D and G, respectively, while F and I are magnified images of the MAA in D and G, respectively. Arrows indicate the region with low expression levels of *Cdkn1c*. Asterisks indicate the high expression level of *Cdkn1c* in the MAA region. Yellow dotted lines indicate the border between apical mesenchymal regions with low and high *Cdkn1c* expression levels. Black dotted lines indicate the border between the dental epithelium and dental mesenchyme. (J) Comparison of the *Cdkn1c* expression levels in the FDR of control and *Lhx6*^*-/-*^ mice at PN4.5 by RT-qPCR. Data are represented as mean ± SD. Asterisk indicates p < 0.05. (K-M) Distribution pattern *of Cdkn1c* in *Lhx6*^*-/-*^ mice at PN4.5. Coronal sections of mandibular first molars were analyzed. L shows magnified image of the LAA in K while M shows magnified image of the MAA in K. Arrows indicate region with low expression levels of *Cdkn1c*. Asterisks indicate the high expression level of *Cdkn1c* in the MAA region. Yellow dotted lines indicate the border between apical mesenchymal regions with low and high *Cdkn1c* expression levels. Black dotted lines indicate the border between the dental epithelium and dental mesenchyme. Scale bar:100μm in A, B, D, G, K; 20μm in E, F, H, I, L and M. MAA, middle apical area; LAA, lateral apical area; FDR, furcation development region; NFDR, non-furcation development region.

Cell proliferation, apoptosis, and epithelium-mesenchyme interaction are all fundamental to patterning and morphogenesis throughout the body [31]. To investigate how Lhx6 regulates tooth root patterning and morphogenesis, we assessed its involvement in these cellular events. We found that loss of Lhx6 adversely affected mesenchymal cell proliferation within the apical region of the dental mesenchyme (Fig 4A–4C) without affecting mesenchymal apoptosis (S4A and S4B Fig). In control mice, apical dental mesenchymal cells in both the LAA and MAA showed active proliferation at PN7.5 (Fig 4A), whereas in *Lhx6*^*-/-*^ mice, actively proliferating Ki67+ mesenchymal cells were mainly located in the LAA (Fig 4B) and mesenchymal cell proliferation was severely reduced in the MAA (Fig 4B). In addition, we found that the distribution pattern of proliferating mesenchymal cells in the FDR of *Lhx6*^*-/-*^ mice at PN7.5 was more similar to the pattern seen in the NDFR, rather than the FDR, of control mice at the same stage (Fig 1E/m/).

Cell proliferation is controlled by the cell cycle, which is negatively regulated by cyclin-dependent kinase inhibitors (CKIs) [32]. A previous study has shown that the CKI Ckdn1c is negatively regulated by Lhx6 during palatogenesis [33]. However, the expression pattern of *Ckdn1c* and whether its expression is regulated by Lhx6 during tooth root development are not known. To answer these questions, we analyzed the distribution pattern of *Ckdn1c* transcripts in control mice and compared the expression of *Cdkn1c* between control and *Lhx6*^*-/-*^ mice. We noticed that the expression patterns of *Cdkn1c* in the FDR and NFDR were different in control mice (Fig 4D–4I). Specifically, the expression levels of *Cdkn1c* in both the LAA and MAA of the FDR were almost undetectable in control mice (Fig 4D and 4F). However, in the NFDR of control mice, *Cdkn1c* mRNA expanded into the MAA but not into the LAA (Fig 4G–4I). We also found that the expression level of *Cdkn1c* was increased in the FDR of *Lhx6*^*-/-*^ mice compared to wild type mice (Fig 4J), suggesting that Lhx6 is also a negative regulator of *Cdkn1c* in the FDR during tooth root development. We further found that the distribution pattern of *Cdkn1c* transcripts was altered in the FDR of *Lhx6*^*-/-*^ mice (Fig 4K–4M), and was similar to the pattern seen in the NFDR of control mice (Fig 4G–4I).

We also learned that the expression pattern of *Cdkn1c* was complementary and mutually exclusive to the distribution pattern of proliferating dental mesenchymal cells in the FDR of both *Lhx6*^*-/-*^ and control mice (Fig 4A and 4B, 4D and 4F and 4K–4M), as the region with low *Cdkn1c* expression level showed high mesenchymal cell proliferation activity and vice versa. These distribution patterns of *Cdkn1c* transcripts and proliferating dental mesenchymal cells supported the notion that *Cdkn1c* is a negative regulator of dental mesenchymal proliferation, and that Lhx6 may regulate mesenchymal proliferation through inhibiting the expression of *Cdkn1c*.

Although *Lhx6* is exclusively expressed in the dental mesenchyme, HERS failed to fuse and the tips of HERS did not dissociate in the *Lhx6*^*-/-*^ mice at PN8.5 (S4C–S4F Fig), suggesting a secondary defect in the dental epithelium as a result of loss of mesenchymal Lhx6.

### Lhx6 coordinates dental mesenchymal progenitor cell differentiation via regulating canonical Wnt signaling pathway

**Fig 5:**
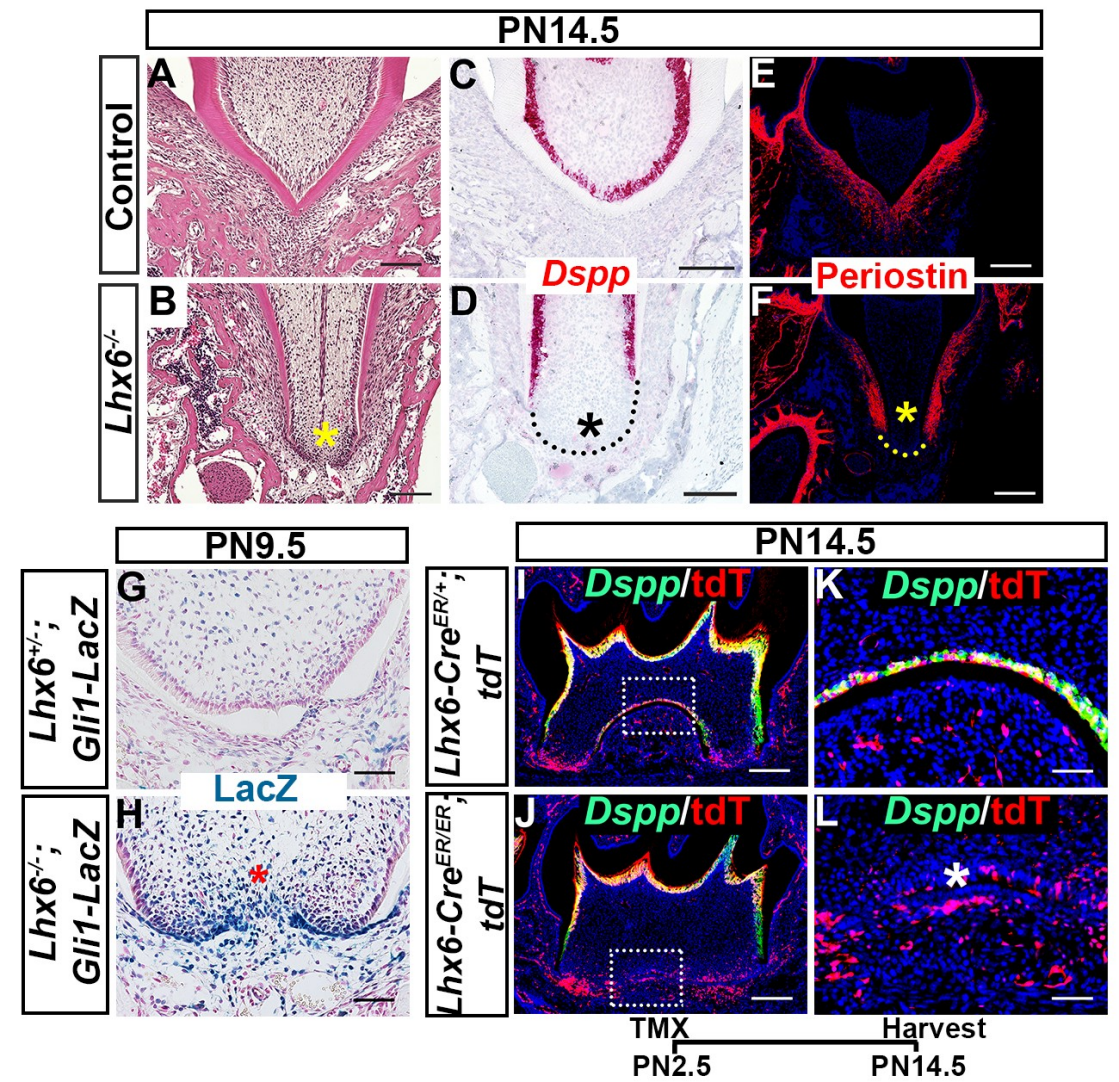
*Lhx6* coordinates dental mesenchymal differentiation. (A-B) H&E staining, (C-D) *in situ* RNAscope assay of *Dspp*, (E-F) immunofluorescence staining of periostin, and (G-H) X-gal staining of FDR from control and *Lhx6*^*-/-*^ mice at indicated stages. Coronal sections of mandibular first molars were analyzed. (I-L) Co-localization of *Dspp* (green) and tdTomato (red) in mandibular first molars of *Lhx6-Cre*^*ER/+*^*;tdT* (control mice with tdTomato reporter) and *Lhx6-Cre*^*ER/ER*^*;tdT* (*Lhx6*^*-/-*^ mice with tdTomato reporter) mice at PN14.5 after tamoxifen injection at PN2.5. Sagittal sections were analyzed. K and L are magnified images of the boxes in I and J, respectively. Asterisks in B, D, F, H, and L indicate where the most significant differences were observed between control and *Lhx6*^*-/-*^ mice. Scale bars: 200μm in I, J; 100μm in A, B, C, D, E, F; 50μm in G, H, K, and L. The schematic at the bottom indicates the induction protocol. TMX: tamoxifen.

Dentin bridge formation by odontoblasts in the MAA is critical for furcation development. We therefore evaluated the odontoblast differentiation of dental mesenchymal cells in the FDR of *Lhx6*^*-/-*^ mice compared to controls. We confirmed that loss of *Lhx6* disturbed the dentin bridge formation (Fig 5A and 5B) and odontoblast differentiation of dental mesenchymal cells specifically in the MAA of the FDR using *Dspp* as an odontoblast differentiation marker (Fig 5C and 5D). We also detected compromised periodontal ligament (PDL) differentiation in the same area using periostin as a PDL differentiation marker (Fig 5E and 5F). In parallel, we found that Gli1+ cells were undetectable in the FDR of control mice at PN9.5 (Fig 5G) while they persisted in the FDR of *Lhx6*^*-/-*^ mice (Fig 5H). Combined with the finding that Gli1+ cells residing in the FDR disappeared after PN9.5 while those located in the NFDR persisted (S5 Fig), these data suggested that the apical dental mesenchyme cells in the presumptive FDR of *Lhx6*^*-/-*^ mice behave as in the NFDR.

Based on our data showing that Lhx6+ cells contribute to the odontoblasts at the root furcation, we asked whether the odontoblast differentiation defect caused by loss of *Lhx6* was cell-autonomous or not. We compared the co-localization patterns of tdTomato and *Dspp* in *Lhx6-Cre*^*ER/ER*^*;tdT* (*Lhx6*^*-/-*^ mice with tdTomato reporter) and *Lhx6-Cre*^*ER/+*^*;tdT* mice (*Lhx6*^*+/-*^ mice with tdTomato reporter, which didn’t show any abnormal furcation phenotype). In the *Lhx6-Cre*^*ER/+*^*;tdT* mice, Lhx6*+* cells contributed to the *Dspp+* cells in the furcation. However, the progeny of labelled Lhx6*+* cells did not express *Dspp* and did not differentiate into odontoblasts in the furcation of *Lhx6-Cre*^*ER/ER*^*;tdT* mice (Fig 5I–5L), suggesting that loss of Lhx6 changed the odontogenic fate of the progenitor cells it labels in a cell-autonomous manner.

**Fig 6:**
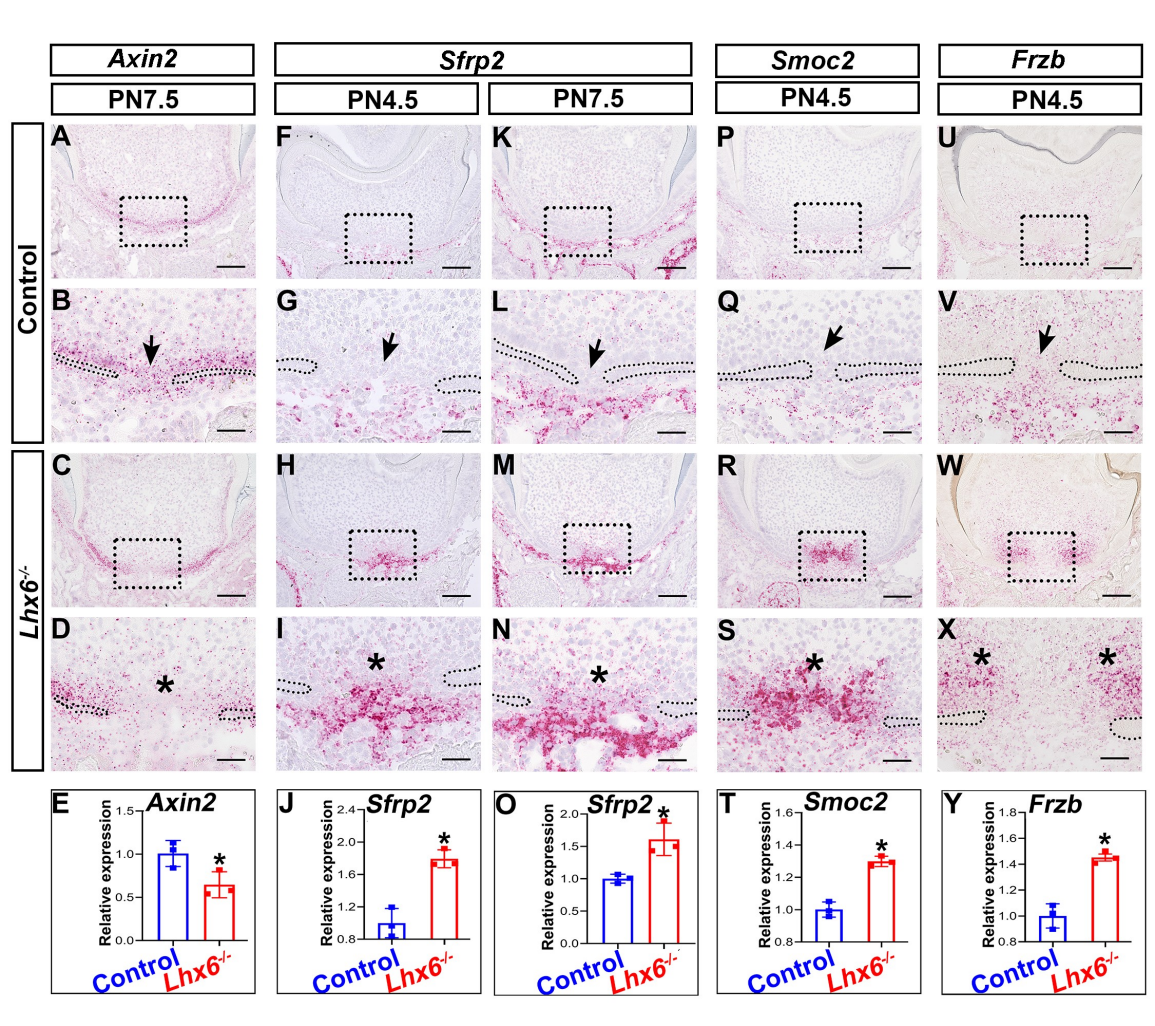
Altered spatial expression patterns and levels of putative downstream targets of *Lhx6*. *In situ* RNAscope assays of *Axin2* (A-D), *Sfrp2* (F-I, K-N), *Smoc2* (P-S) and *Frzb* (U-X) in the FDR of control and *Lhx6*^*-/-*^ mice at indicated stages. Coronal sections of mandibular first molars were analyzed. Quantification of the expression levels of *Axin2* (E) and *Sfrp2* (O) between control and *Lhx6*^*-/-*^ mice at PN7.5, and comparison of *Sfrp2* (J), *Smoc2* (T) and *Frzb* (Y) at PN4.5 using RT-qPCR. Data are represented as mean ± SD. Boxes in A, C, F, H, K, M, P, R, U and W are shown magnified in B, D, G, I, L, N, Q, S, V and X, respectively. The arrow in B indicates the high *Axin2* expression level in the MAA of control mice, and arrows in G, L, Q, and V indicate the low gene expression levels in the MAA of control mice. Asterisks in D, I, N, S, and X indicate the region where the most significant differences were observed between control and *Lhx6*^*-/-*^ mice. Asterisks in E, J, O, T, and Y indicate p < 0.05. Scale bars: 100μm in A, C, F, H, K, M, P, R, U and W; 20μm in B, D, G, I, L, N, Q, S, V and X.

To understand how Lhx6 regulates odontoblast differentiation, we used laser-capture microdissection to collect the apical part of the FDR of mandibular first molars from both *Lhx6*^*-/-*^ and control mice for RNAseq. We harvested samples at PN4.5 based on our previous finding that crown development finishes and root development initiates at this stage [11], as well as our observation that the cellular dynamics in the FDR and NFDR diverged after this stage. Hierarchical clustering showed that gene expression profiles of *Lhx6*^*-/-*^ and control mice were well separated (S6A Fig). We identified 284 differentially expressed genes (DEGs, ≥ 1.5-fold, p < 0.05), of which 120 were upregulated and 164 were downregulated (S6A Fig). Pathway analysis using PANTHER classification system revealed that these DEGs belonged to several signaling pathways including Wnt, Cadherin, and Integrin (S6B Fig). Wnt signaling pathway was the top pathway affected by *Lhx6* loss (S6B Fig). Canonical Wnt signaling pathway plays important roles in regulating root formation [5]. We therefore narrowed our focus to this pathway. Using *Axin2* as a readout [34], we found that the activity of canonical Wnt pathway was down-regulated in the FDR of the *Lhx6*^*-/-*^ mice, especially in the MAA (Fig 6A–6E).

To investigate the possible mechanisms by which Lhx6 regulates the Wnt signaling pathway, we next analyzed the expression patterns of the top 15 DEGs (S1 Table). In control mice, three DEGs, including *Smoc2*, *Sfrp2* and *Frzb (Sfrp3)*, showed spatially specific expression patterns that differed between the FDR and NFDR (S6C–S6N Fig). These three genes had low expression levels in both the MAA and LAA of the FDR (S6C, S6E, S6G, S6I, S6K and S6M Fig). However, the expression levels of *Smoc2* and *Sfrp2* were high in the MAA of the NFDR (S6D, S6F, S6H and S6J Fig) while *Frzb* transcripts were mainly enriched in the LAA of the NFDR (S6L and S6N Fig). We further validated the expression changes of *Smoc2*, *Sfrp2* and *Frzb* in *Lhx6*^*-/-*^ mice as compared to control mice by *in situ* RNAscope and RT-qPCR. We found that all three of these Lhx6 putative target genes showed increased expression levels in the FDR of *Lhx6*^*-/-*^ mice (Fig 6F–6Y), which is highly consistent with the RNAseq results. We also found that loss of *Lhx6* disrupted the expression patterns of *Smoc2*, *Sfrp2* and *Frzb* in the FDR, and the expression patterns of these three genes in the FDR of *Lhx6*^*-/-*^ mice (Fig 6F–6I, 6K–6N, 6P–6S and 6U–6X) were similar to their expression patterns in the NFDR of control mice (S6C–S6N Fig).

**Fig 7:**
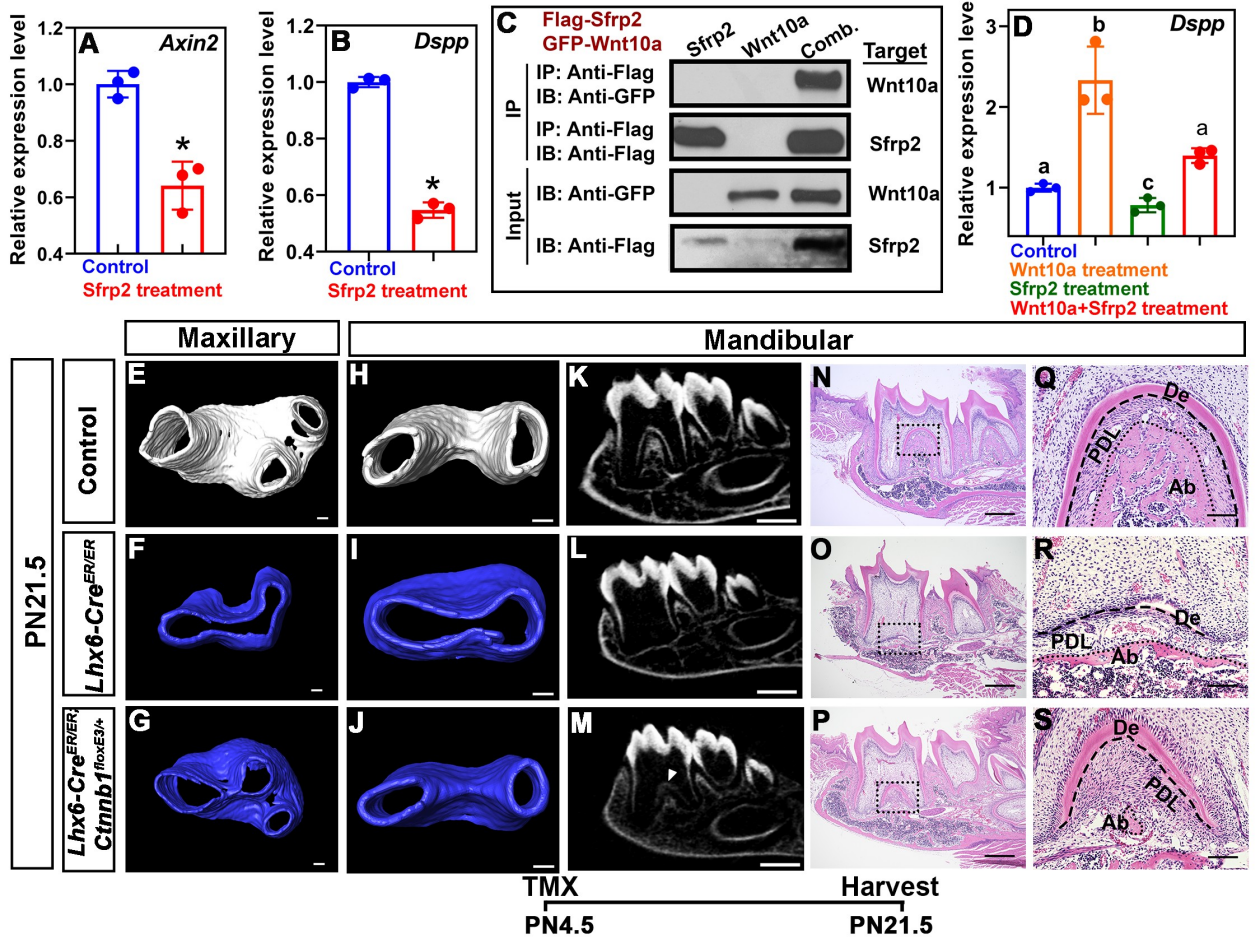
*Lhx6* regulates dental mesenchymal cell differentiation through regulating canonical Wnt signaling pathway by inhibiting Sfrp2. (A-B) RT-qPCR assay for *Axin2* (A) and *Dspp* (B) in cultured dental pulp cells from PN2.5–3.5 control mice with or without recombinant Sfrp2 treatment. Data are represented as mean ± SD, and asterisks indicate p < 0.05. (C) Co-immunoprecipitation using Flag-tagged Sfrp2 and GFP-tagged Wnt10a expressed in 293T cells. Sfrp2 was immunoprecipitated (IP) and Wnt10a was immunoblotted (IB) for the physical interaction analysis. Comb.: simultaneous transfection with Flag-tagged Sfrp2 and GFP-tagged Wnt10a vectors. (D) RT-qPCR for *Dspp* in cultured dental pulp cells from PN2.5–3.5 control mice treated with recombinant Sfrp2, Wnt10a or their combination. Data are represented as mean ± SD. Different lowercase letters above bars indicate statistically significant intergroup differences (p < 0.05), and same lowercase letters above bars indicate no statistically significant intergroup differences (p > 0.05). (E-G) microCT images of the maxillary first molars of PN21.5 control (E), *Lhx6*^*-/-*^ (F) and *Lhx6*^*-/-*^ rescue (*Lhx6-Cre*^*ER/ER*^*;Ctnnb1*^*floxE3/+*^; G) mice after tamoxifen injection at PN4.5. (H-M) microCT images of the mandibular first molars of PN21.5 control (H, K), *Lhx6*^*-/-*^ (I, L) and *Lhx6*^*-/-*^ rescue (*Lhx6-Cre*^*ER/ER*^*;Ctnnb1*^*floxE3/+*^; J, M) mice after tamoxifen injection at PN4.5. Arrowhead in M indicates the furcation of rescue mice. (N-S) H&E staining of mandibular first molars of PN21.5 control (N, Q), *Lhx6*^*-/-*^ (O, R) and *Lhx6*^*-/-*^ rescue (*Lhx6-Cre*^*ER/ER*^*;Ctnnb1*^*floxE3/+*^; P, S) mice after tamoxifen injection at PN4.5. Sagittal sections of mandibular first molars were analyzed. Q, R and S are magnified images of the boxes in N, O and P, respectively. Dashed lines in Q, R and S indicate the border between tooth hard tissues and periodontal ligament, while dotted lines in Q, R and S indicate the border between periodontal ligament and alveolar bone. The schematic at the bottom indicates the induction protocol. De, dentin; PDL, periodontal ligament; Ab, alveolar bone. Scale bars: 100μm in E-J and Q-S; 500μm in K-P. TMX: tamoxifen.

Previous studies have shown that Sfrp2 is a Wnt antagonist [35,36]. In the *Lhx6*^*-/-*^ mice, *Sfrp2* transcripts were enriched in the MAA (Fig 6H, 6I, 6M and 6N) where the decreased expression of *Axin2* was mainly detected (Fig 6C and 6D). Therefore, we further narrowed our focus to Sfrp2 as a potential regulator through which Lhx6 regulates the activity of Wnt signaling pathway. To test this hypothesis, we first cultured mouse dental mesenchymal cells as described in our previous study [7] and then treated them with mouse recombinant Sfrp2 protein. We found that Sfrp2 down-regulated the activity of canonical Wnt signaling pathway, as revealed by the expression of *Axin2* (Fig 7A). Wnt signaling pathway regulates odontoblast differentiation, such that inactivating canonical Wnt signaling pathway blocks odontoblast differentiation and results in complete ablation of root dentin formation [37,38]. In cell culture, we also found that supplementation of Sfrp2 inhibited odontoblast differentiation, as revealed by decreased *Dspp* expression (Fig 7B). Briefly, these data suggested that Sfrp2 is among the key regulators through which Lhx6 controls the activity of Wnt signaling pathway as well as odontoblast differentiation during furcation development. Because Sfrp2 can mediate Wnt signaling through binding to Wnt ligands [35,36], we sought to determine which Wnt ligands were affected by Sfrp2. We found that four canonical Wnt ligands were expressed during postnatal root development, namely *Wnt10a*, *Wnt6*, *Wnt4* and *Wnt3a*, although the expression levels of these ligands did not significantly change between control and *Lhx6*^*-/-*^ mice (S7 Fig). Previous studies showed that Sfrp2 is able to bind to Wnt6, Wnt4 and Wnt3a, and mediates their induction of Wnt signaling [35,36,39]. It remains unclear whether Sfrp2 also interacts with Wnt10a. Loss of Wnt10a results in a similar furcation phenotype to that observed in *Lhx6*^*-/-*^ mice [17,18], and Wnt10a can induce the expression of *Dspp* [40]. We therefore investigated the potential interaction between Sfrp2 and Wnt10a. Using co-immunoprecipitation, we found Wnt10a can physically bind to Sfrp2 (Fig 7C). In addition, treating cells with mouse recombinant Wnt10a protein was able to rescue the compromised odontoblast differentiation caused by Sfrp2 (Fig 7D), suggesting that the increased Sfrp2 in *Lhx6*^*-/-*^ mice may antagonize the function of Wnt10a and contribute to the furcation phenotype.

To further validate that the downregulation of canonical Wnt activity in the Lhx6+ lineage is involved in the odontoblast differentiation and furcation defects of *Lhx6*^*-/-*^ mice, we generated a *Lhx6-Cre*^*ER/ER*^*;Ctnnb1*^*floxE3/+*^ compound mutant mouse model in which, in addition to loss of Lhx6 expression, Wnt signaling activity is constitutively activated in the Lhx6+ cell derivatives after tamoxifen induction at PN4.5. We first analyzed the roots of double heterozygous compound mutants (i.e., *Lhx6-Cre*^*ER/+*^*;Ctnnb1*^*floxE3/+*^), and found there was no abnormal furcation phenotype (S8 Fig). We found that furcation development was partially rescued in the compound mutant mice (Fig 7E–7M). Histological analysis detected dentin and organized odontoblasts at the furcation, although it was situated more apically than in wild type controls (Fig 7N–7S). PDL development, though not alveolar bone development, was also rescued (Fig 7N–7S). These data supported our hypothesis that down-regulated Wnt activity in the Lhx6+ lineage is among the key factors underlying odontoblast differentiation and furcation defects in *Lhx6*^*-/-*^ mice.

## Discussion

**Fig 8:**
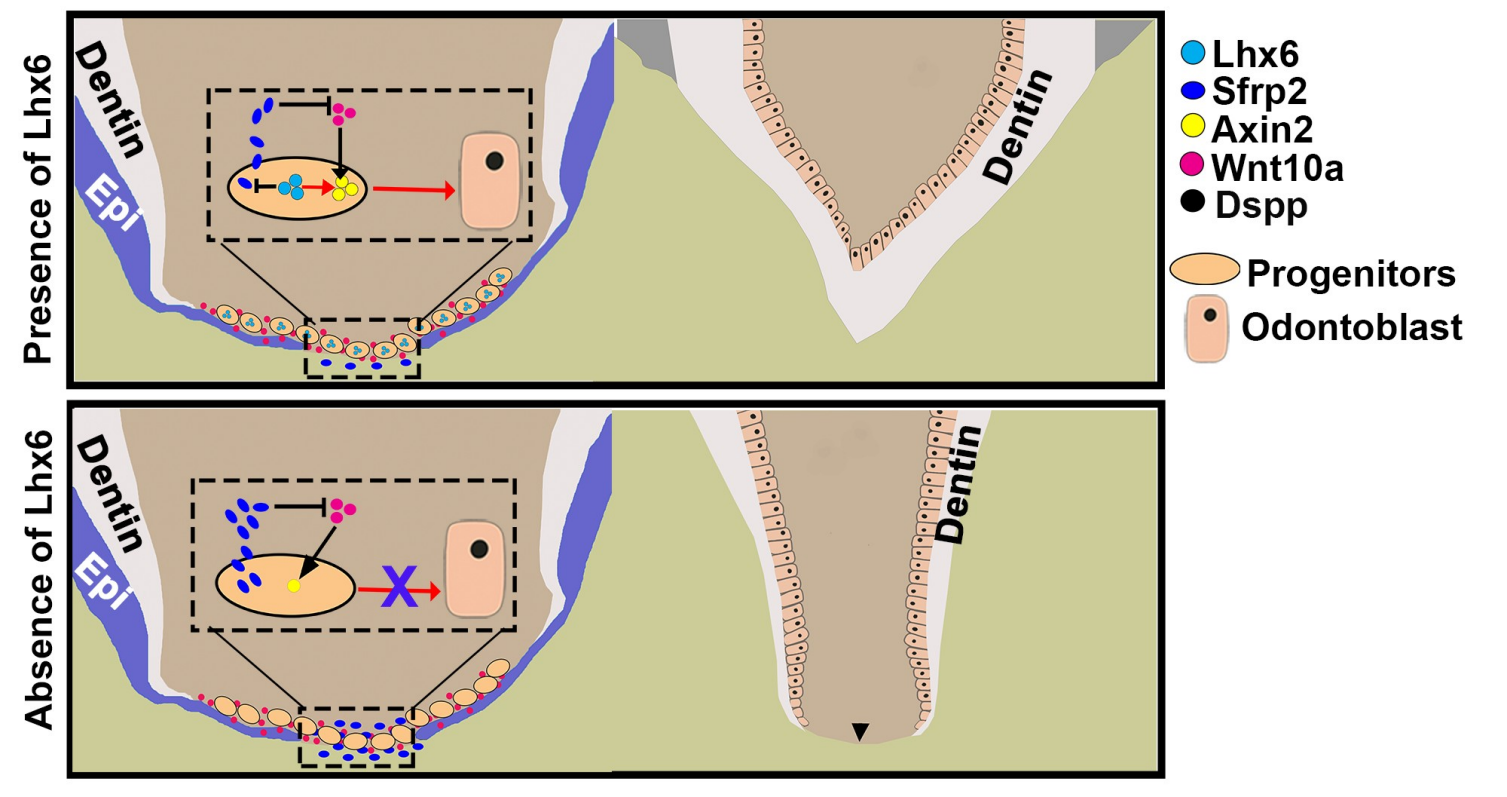
Schematic drawing of the role of *Lhx6* in regulating root furcation development. Lhx6 labels a group of progenitor cells that contribute to the odontoblasts and dentin bridge in the furcation. In these progenitors, Lhx6 is critical for activating the canonical Wnt signaling pathway, which in turn is important for the differentiation of Lhx6+ progenitor cells into odontoblasts. One mechanism through which Lhx6 mediates the canonical Wnt signaling pathway is by inhibiting the expression of Wnt antagonist Sfrp2, thereby promoting the role of Wnt10a in inducing odontoblast differentiation. In the absence of Lhx6, the progenitors residing in the middle apical area (MAA) have significantly decreased canonical Wnt signaling activity, so they cannot differentiate into odontoblasts and form the dentin bridge. One important reason is that without Lhx6, the expression level of Sfrp2 in the MAA increases sharply, and the increased Sfrp2 antagonizes the induction by Wnt10a. The arrowhead indicates absence of furcation. Epi: epithelium.

Improved understanding of the mechanisms controlling tooth development and morphogenesis could help to reveal the common principles that orchestrate organogenesis throughout the body. In the present study, we identified a previously unknown subpopulation of Gli1+ mesenchymal progenitor cells closely associated with furcation development (Fig 8). We further revealed that Lhx6 is expressed in these progenitors and is required for their odontoblast differentiation in a cell-autonomous manner. In the absence of *Lhx6*, the dentin bridge formation in the furcation is disrupted, resulting in the transformation of multi-rooted teeth into single-rooted ones (Fig 8). Specifically, we found that Lhx6 maintains the activity of canonical Wnt signaling pathway to control the lineage commitment of these progenitor cells, and one important mechanism through which Lhx6 mediates the canonical Wnt signaling pathway is by regulating Wnt antagonist Sfrp2 (Fig 8).

The heterogeneity of stem/progenitor cells within specific tissues is increasingly recognized [41,42] as it has been revealed by their diverse transcriptional profiles, cellular morphology, proliferation and differentiation potential [42–44]. Gli1+ mesenchymal cells have been identified as a critical cell population supporting mouse tooth root growth [7,11]. Gli1 marks a heterogeneous cell population in the dental mesenchyme including both niche cells and root progenitor cells. Specifically, Gli1+;Runx2+ cells are niche cells that regulate root elongation in mouse molars [45]. In the present study, we have identified Lhx6+ cells in the Gli1+ progenitor cell pool. Lhx6+ cells give rise to various differentiated dental mesenchymal cell types *in vivo* including odontoblasts, pulp cells and periodontal cells. Although they can also be characterized as progenitor cells, Lhx6+ cells make a limited contribution in comparison to the entire Gli1+ progenitor cell population, and not all Gli1+ progenitor cells express Lhx6. These findings provide evidence for transcriptional heterogeneity in adult stem/progenitor cell subpopulations, consistent with what has been reported previously in myeloid progenitors [46] and neural stem cells [47]. Intriguingly, the progeny of Lhx6+ cells show a distinct spatial distribution: Lhx6+ derivatives are predominantly located at and near the furcation with limited distribution in the root mesenchyme away from the furcation. This preferential contribution of Lhx6+ derivatives to a specific region suggests that distinct subpopulations exist within the mouse root stem/progenitor cell pool, each of which is closely associated with formation of a specific anatomical structure. The spatial distribution of Lhx6+ derivatives during root development therefore provides a new perspective on the heterogeneity of stem/progenitor cells.

We show that cellular dynamics and molecular regulation differ in the apical portions of the FDR and NFDR. This finding was enabled by our separate analyses of defined sub-regions including the MAA and LAA in both the FDR and NFDR. The division between the MAA and LAA is supported by both cellular (e.g. mesenchymal proliferation and differentiation activity) and molecular evidence (e.g. expression patterns of genes such as *Smoc2*, *Ckdn1c*, *Frzb* and *Sfrp2*). We further demonstrated that Lhx6 is indispensable for maintaining the specific character of the FDR, as loss of Lhx6 disrupts both the cellular and molecular patterning of the FDR, making them more similar to those of the NFDR. As a result, no furcation formed in *Lhx6*^*-/-*^ mice. Although *Lhx6* is broadly expressed in the mesenchyme of the MAA and LAA in the FDR, it precisely regulates target genes in distinct ways within these sub-regions. For example, loss of Lhx6 mainly increases the expression of *Cdkn1c*, *Smoc2* and *Sfrp2* in the MAA of the FDR, but it increases the expression of *Frzb* predominantly in the LAA of the FDR. How Lhx6 achieves this regional functional specificity in regulating root furcation development will require additional study. Our data suggest that Lhx6 inhibits mesenchymal cell proliferation via *Cdkn1c* and suppresses mesenchymal cell differentiation via *Sfrp2*. Previous studies have highlighted the altered mesenchymal cell proliferation in transgenic mice with furcation defects [17,48,49]. We emphasize the importance of mesenchymal cell differentiation, especially progenitor cell commitment, as an important process in furcation formation in this study. We identified Sfrp2 as a potential target through which Lhx6 regulates mesenchymal differentiation. Sfrp2 is a member of the secreted frizzled-related protein (Sfrp) family. Loss of Sfrp2 leads to brachydactyly, mild mesomelic shortening and posterior soft tissue syndactyly [50]. Sfrp2 disrupts myogenic differentiation of mouse embryonic stem cells by regulating *Wnt3a* transcription [51]. We show that, during odontogenesis, Sfrp2 physically interacts with Wnt10a, blocks its function, and downregulates the activity of canonical Wnt signaling pathway. In ATAC-seq performed on mouse molars by our group (Wen et al., 2020[45], GSE151563), we did not detect an Lhx6 binding motif in the proximal regulatory regions (< 50Kb) upstream or downstream of the *Sfrp2* gene locus. However, there are some potential Lhx6 binding sites in the distal region (S9 Fig). A previous study showed that Lhx6 can mediate gene expression by binding and activating enhancers [23]. It will be very interesting to test in the future whether these potential binding sites are enhancers or repressors of *Sfrp2*, and whether Lhx6 can bind to these sites to regulate *Sfrp2* expression.

During mammalian development, cell fate determination and tissue morphogenesis are precisely coordinated [52]. Accumulating studies have shown that progenitor/stem cell fate specification is involved in aspects of tooth root morphogenesis. For instance, loss of BMP-Smad4 signaling prevents the disappearance of Sox2+ epithelial stem cells, leading to a lack of crown-to-root transition and the absence of tooth root formation [6]. In addition, the fate regulation of Gli1+ mesenchymal progenitor cells by BMP signaling is crucial for tooth root elongation [7]. In this study, we learned that disrupting the odontoblast fate commitment of progenitor cells by deleting *Lhx6* causes failure of furcation formation and root number patterning defect. These studies also collectively demonstrate that tooth root development provides an excellent model with which to answer questions concerning how specific signaling cascades link progenitor/stem cell fate commitment to control 3D tissue morphology. Intriguingly, as very recently reported in *Osr2-Cre;Ezh2*^*fl/fl*^ mice, a disturbance to root number patterning does not appear to significantly affect tooth root length [1]. The same tooth root patterning phenotype is observed in *Lhx6*^*-/-*^ mice in the present study. Other studies have shown shortened root phenotypes in certain other mutant mouse models, including *Gli1-Cre*^*ER*^*;SmoM2*^*fl/fl*^ [11] and *Gli1-Cre*^*ER*^*;Runx2*^*fl/fl*^ [45]; however, the molars of these mice have normal numbers of roots. These data suggest that the root length and root number determination of mouse molars are largely independent after the crown-to-root transition is successfully initiated.

It is well known that crown patterning and morphogenesis are initially regulated by signals from the dental epithelium and subsequently depend on continuous and reciprocal epithelial-mesenchymal interactions [53]. The location of the signaling center that ultimately determines tooth root patterning and morphogenesis has remained elusive. The mesenchyme cannot be overlooked as a potential source of regulatory signals, since it has instructive functions even in the development of epithelial organs [54]. We found that *Lhx6*, a gene specifically and consistently expressed in the dental mesenchyme, regulates the patterning of the tooth roots, strongly supporting our previous finding that mesenchymal regulation plays a dominant role in patterning tooth root morphology [1]. Some recently published studies have shown that deletion of Wnt10a in the dental epithelium leads to an absence or apical displacement of the root furcation in mouse molars [17,18]. However, we showed that the furcation defect occurs without necessarily influencing the expression of Wnt10a in *Lhx6*^*-/-*^ mice. In addition, we found that the function of Wnt10a can be blocked by mesenchymal Sfrp2. These data support the notion that the dental mesenchyme is a signaling center for root patterning and morphogenesis.

In conclusion, the present study has revealed previously unknown heterogeneity among tooth root progenitor cells; uncovered how Lhx6 precisely regulates the patterning of the tooth root, especially progenitor cell fate commitment; and revealed the importance of dental mesenchymal regulation for root morphogenesis. Collectively, these findings represent an important contribution to our understanding of the mechanisms that guide organogenesis. These findings may also serve to illustrate developmental processes shared with other organs while providing important information for future tooth root regeneration strategies.

## Material and methods

### Ethics statement

All animal studies were approved by the Institutional Animal Care and Use Committee of the University of Southern California. The animals were handled according to approved IACUC protocol #11765 at the University of Southern California.

### Animals

C57BL/6J and transgenic mouse lines were used, including *Lhx6-Cre*^*ER*^ (JAX#010776, The Jackson Laboratory) [28], *ROSA26*^*loxP-STOP-loxP-tdTomato*^ (*tdTomato* conditional reporter, JAX#007905, The Jackson Laboratory) [55], *Gli1-LacZ* (JAX#008211, The Jackson Laboratory) [56], *Gli1-Cre*^*ER*^ (JAX#007913, The Jackson Laboratory) [57] and *Ctnnb1*^*floxE3*^ [58]. All mice were housed in pathogen-free conditions, and newborn pups were documented as postnatal stage PN0.5. Both male and female mice at indicated stages were collected, genotyped and analyzed.

### Tamoxifen administration

For Cre^ER^ activation, tamoxifen (Sigma, T5648) dissolved in corn oil (Sigma, C8267) with a final concentration of 20mg/ml was injected intraperitoneally (10μL/1g body weight, single injection unless otherwise specified) at PN2.5 for lineage tracing. To activate the expression of *Ctnnb1* in the Lhx6+ cell lineage, the tamoxifen dosage was adjusted to 8μL/1g body weight, and the tamoxifen was injected at PN4.5.

### microCT analysis

Both mandibles and maxillae were dissected under a stereomicroscope (Leica L2) and fixed in 4% paraformaldehyde (PFA) overnight at room temperature. microCT scanning was carried out with Skyscan 1174v1.2 (Bruker Corporation, USA) at the Center for Craniofacial Molecular Biology, University of Southern California. Images at a resolution of 16.7μm were acquired with the X-ray source at 50kVp and 800μA. The reconstruction of 3D images was completed using Avizo/Amira 9.5.0 (FEI Visualization Sciences Group, France).

### Histology assay

Mouse mandibles were dissected and fixed in 10% neutralized buffered formalin (NBF, Sigma) overnight at room temperature, then decalcified with 10% EDTA in 1xPBS for 1–14 days based on mouse age. Decalcified mandibles were dehydrated and embedded in paraffin. Tissue blocks were sectioned at 5μm using a microtome (Leica) and mounted on SuperFrost Plus slides (Fisher). Hematoxylin and Eosin (H&E) staining was completed following standard protocols.

### Immunofluorescence assay

Immunofluorescence assays were performed on paraffin-embedded sections prepared as described above. Sections were dried for 1 hour at 55°C, deparaffinized and rehydrated. After antigen retrieval (Vector, H-3300), sections were blocked for 1 hour at room temperature in blocking solution (PerkinElmer, FP1020), and then incubated with primary antibodies diluted in blocking solution at 4°C overnight. After washing three times with PBST (0.1% Tween20 in 1xPBS), sections were incubated with secondary antibodies at room temperature for 1 hour. For periostin and Lhx6 protein detection, fluorescent signal amplification Tyramide SuperBoost Kits, including goat anti-rabbit Alexa Fluor-647 (B40926, Thermo Fisher Scientific) and goat anti-mouse Alexa Fluro-488 (B40912, Thermo Fisher Scientific) were used according to the supplier’s protocols. DAPI (Sigma, D9542) was used for nuclear staining. Detailed information on antibodies used in this study is available in S2 Table.

### TUNEL assay

Specimens were harvested, fixed, decalcified, and sectioned as described for immunofluorescence assays. Apoptotic cells were detected using Click-it Plus TUNEL Assay for *In Situ* Apoptosis Detection kit (Thermo Fisher, C10617) following the manufacturer’s recommended protocol.

### *In situ* RNAscope assay

Tissues were fixed in 10% NBF overnight at room temperature, decalcified with DEPC-treated 10% EDTA in 1×PBS at 4°C, dehydrated sequentially with 15% sucrose in 1xPBS (4°C, overnight) and 30% sucrose in 1xPBS/OCT (4°C, overnight), and embedded in OCT (Sakura, Tissue-Tek, 4583) on liquid nitrogen immediately. Frozen tissue blocks were sectioned at 8μm on a cryostat (Leica CM3050S) and mounted on SuperFrost Plus slides. *In situ* RNA expression detection was performed using RNAscope 2.5 HD Assay-RED kit (Advanced Cell Diagnostics, 322350) and RNAscope 2.5 HD Multiplex Fluorescent v2 (Advanced Cell Diagnostics, 323110) according to the manufacturer’s instructions. Probes used for *Lhx6*, *Lhx8*, *Dspp*, *Axin2*, *Sfrp2*, *Frzb* (*Sfrp3)*, *Wnt3a*, *Wnt4*, *Wnt6 and Wnt10a* were designed and synthesized by Advanced Cell Diagnostics. Detailed information on probes is provided in S3 Table.

### X-gal staining

The mandibles were fixed in 0.2% glutaraldehyde overnight in 4°C, decalcified in 10% EDTA supplemented with 2mM MgCl_2_. The samples were dehydrated through a sucrose series and embedded in OCT. To detect β-galactosidase (β-gal) activity in tissue sections, cryosections were stained in X-gal staining solution for 3–4 h at 37°C in the dark according to standard procedure [7].

### Laser capture microdissection and RNA-sequencing

First mandibular molars were collected from both control and littermate mutant mice at PN4.5. After dissection, the mandibles were washed once in pre-chilled DEPC-treated 1xPBS and transferred into a mold filled with pre-chilled OCT and frozen using liquid nitrogen. Next, samples were sectioned into slices of 12μm thickness on a cryostat and mounted on Polyethylenenaphthalate (PEN) membrane slides (Zeiss). The sections were fixed and stained with H&E staining strictly following the manufacturer’s protocol. We performed laser capture microdissection of the apical tissues at the furcation development region of both control and mutant mice using the Zeiss PALM Laser Capture Microdissection System. To get sufficient total RNA, tissues from eight mandibular first molars were combined together to prepare one RNA library. The total RNA was isolated using an RNeasy Plus Micro Kit (Qiagen, 74034), and the quality of RNA was determined using an Agilent 2100 Bioanalyzer. The cDNA library preparation and sequencing were performed at the UCLA Technology Center for Genomics & Bioinformatics. Single-end reads with 75 cycles were performed on the Illumina Nextseq 500 platform for 3 pairs of samples. Raw reads were trimmed, aligned to the mm10 genome using STAR (version 2.6.1d), and normalized using RPKM. Differential expression was calculated by selecting transcripts with fold change ≤ -1.5 or ≥ 1.5 and a significance level of p < 0.05. The pathway analysis was done using PANTHER classification system [59].

### Cell culture, recombinant protein treatment

Dental apical mesenchymal cells of the mandibular first molars of PN2.5 to PN3.5 mice were dissected, cut into small pieces, and seeded into 24-well plate culture dishes (Corning) with α-MEM supplemented with 20% FBS, 100U/ml penicillin and 100μg/ml streptomycin (Life Science Technologies). For each well, at least 6 first mandibular molar tooth germs were required to get sufficient cells. The cells were cultured without disturbance for 24h to allow initial cell attachment, then fresh medium was added and the cells were cultured until 80% confluent. Mouse recombinant Sfrp2 protein (R&D) and Wnt10a (LsBio) with a final concentration of 2.5μg/ml were dissolved in odontoblast differentiation medium (1% FBS, 5mM β-glycerophosphate (β-GP, Sigma, G9422), 50μg/ml ascorbic acid (Sigma, A4403), and 10nM dexamethasone (DEX, Sigma, D4902), and added into each well. The cells were harvested 7 days after odontogenic induction for RT-PCR assays.

### Tissue dissection, RNA extraction and RT-qPCR

The apical third of the first mandibular molar was dissected out first, and then the middle third of the apical tissue was collected for RNA extraction and RT-qPCR validation. Briefly, the total RNA was isolated using an RNeasy Plus Micro Kit (Qiagen, 74034), and the cDNA was synthesized using an iScript cDNA Synthesis kit (Bio-Rad). The quantitation reaction was done with SsoFast EvaGreen Supermix (Bio-Rad) on a Bio-Rad CFX96 Real-Time System. The primer sequences were obtained from PrimerBank [60] and are listed in S4 Table.

### Co-IP assays

Flag-Sfrp2 (MR204070, OriGene) and GFP-Wnt10a (MG206595, OriGene) vectors were transfected into 293T cells. Cells were harvested 48h after transfection and lysed in lysis buffer (50mM Tris-HCl (pH7.5), 150mM NaCl, 2mM EDTA, 0.1% NP-40, 10% glycerol and protease inhibitor cocktail). Lysates were immunoprecipitated with anti-Flag antibody and protein G-Sepharose 4 fast flow (GE Healthcare). Immune complexes were washed three times with IP buffer and immunoblotted with anti-Flag or anti-GFP antibodies (S2 Table).

### Image acquisition

The Immunofluorescence, TUNEL and RNAscope images were captured using an All-in-one Fluorescence Microscope (Keyence, BZ-X710) at the Center for Craniofacial Molecular Biology, University of Southern California.

### ImageJ image analysis

ImageJ was used to determine the percentage of immunostained area out of the total area. To calculate the percentage of positive immunostaining, selected regions were first converted to 8-bit binary images, and then positive signals were measured with the “Analyze Particles” function. The derived area was then divided by the total area.

### Sample size

We analyzed n≥3 samples for each experimental group for all experiments unless otherwise stated.

### Statistical analysis

Statistical analysis was completed with GraphPad Prism. Significance was assessed by independent two-tailed Student’s t-test or analysis of variance, and the chosen level of significance for all statistical tests in this study was p <0.05.

## Supporting information

S1 Fig Expression pattern of *Lhx6* during tooth crown development. Dotted lines indicate border between dental epithelium and mesenchyme. Epi, epithelium; Mes, mesenchyme. Scale bars: 100μm.
(PDF)

S2 Fig *Lhx6*^*-/-*^ mice show a reduction in body size and unaffected tooth crown morphology. (A-B) *Lhx6*^*-/-*^ mice were born alive and appeared grossly normal in the first week after birth, but developed an obvious body size reduction and failed to survive past 1 month. (C-D) Immunofluorescence staining of Lhx6 on coronal mouse molar sections. (E-F) H&E staining of mandibular first molars of control (E) and *Lhx6*^*-/-*^ (F) mice at PN4.5 on sagittal sections. Scale bars: 100μm in C and D; 200μm in E and F.
(PDF)

S3 Fig Expression patterns of *Lhx6* and *Lhx8* during tooth morphogenesis. RNAscope assays of *Lhx6* and *Lhx8* at indicated stages. Coronal sections were analyzed. Dotted lines indicate border between dental epithelium and mesenchyme. Epi, epithelium; Mes, mesenchyme. Scale bars: 100μm.
(PDF)

S4 Fig Loss of *Lhx6* causes secondary epithelial defects through tissue-tissue interaction. (A-B) TUNEL assays of control and *Lhx6*^*-/-*^ mice at PN7.5 on coronal sections. (C-F) Immunofluorescence staining of epithelial marker Krt14 in control and *Lhx6*^*-/-*^ mice at PN8.5 on coronal sections. Boxes in C and D are shown at higher magnification in E and F, respectively. Asterisk indicates the absence of epithelial fusion and dissociation. Scale bars: 100μm in A-D; 20μm in E-F.
(PDF)

S5 Fig Distribution patterns of Gli1+ cells between FDR and NFDR at indicated stages. (A-H) X-gal staining of coronal sections of *Gli1-LacZ* reporter mouse molars at indicated stages. Coronal sections were analyzed. FDR, furcation development region; NFDR, non-furcation development region. Scale bars: 500μm in A, C, E, G; 50μm in B, D, F, H.
(PDF)

S6 Fig Spatial expression patterns of *Smoc2*, *Sfrp2* and *Frzb* in FDR and NFDR of control mice. (A) Heatmap hierarchical clustering showing the gene expression profiles of FDR in control and *Lhx6*^*-/-*^ mice at PN4.5. (B) Pathway analysis of differentially expressed genes using the PANTHER tool. Only the top ten pathways in which differentially expressed genes were enriched are shown. (C-N) RNAscope assays of *Smoc2* (C-F), *Sfrp2* (G-J) and *Frzb* (K-N) of PN4.5 control mice. Coronal sections were analyzed. Boxes in C, D, G, H, K, and L are shown at higher magnification in E, F, I, J, M and N, respectively. Dotted lines in E, I, M and N indicate border between dental epithelium and mesenchyme. Arrows in E, I and M indicate regions with lower gene expression levels, while asterisks in F, J and N indicate regions with high gene expression levels. Scale bars:100μm in C, D, G, H, K and L; 20μm in E, F, I, J, M and N. FDR, furcation development region; NFDR, non-furcation development region.
(PDF)

S7 Fig No significant difference observed in the expression levels of Wnt ligands between controls and *Lhx6*^*-/-*^ mice. *In situ* RNAscope assays of *Wnt10a* (A-D), *Wnt4* (E-H), *Wnt6* (I-L), and *Wnt3a* (M-P) of control mice and *Lhx6*^*-/-*^ mice at PN4.5. Coronal sections were analyzed. Dotted lines indicate border between dental epithelium and mesenchyme. Boxes in A, C, E, G, I, K, M and O are shown at higher magnification in B, D, F, H, J, L, N and P respectively. Scale bars: 100μm in A, C, E, G, I, K, M and O; 20μm in B, D, F, H, J, L, N and P.
(PDF)

S8 Fig Compound heterozygous mice have no abnormal furcation phenotype. microCT scanning of maxillary and mandibular first molars of PN21.5 control (A-D) and *Lhx6-Cre*^*ER/+;*^*Ctnnb1*^*floxE3/+*^ (E-H) mice. Scale bars: 200μm in A, C, E, G; 100μm in B, F; 80μm in D and H. The schematic at the bottom indicates the induction protocol. TMX: tamoxifen.
(PDF)

S9 Fig ATAC-seq analysis of molars from PN7.5 wild type mice. No Lhx6 binding motif was detected in the proximal regulatory region (<50Kb) upstream or downstream of the *Sfrp2* gene locus. However, there are some potential Lhx6 binding sites in the distal region as indicated by the arrows.
(PDF)

S1 Table Top 15 differentially expressed genes discovered via RNA-sequencing. (PDF)

S2 Table Primary antibody information. (PDF)

S3 Table Probe information. (PDF)

S4 Table Primer information. (PDF)
