# Supplementary material for: Lhx6 regulates canonical Wnt signaling to control the fate of mesenchymal progenitor cells during mouse molar root patterning
Source: PLoS Genet. 2021 Feb 17;17(2):e1009320. doi: 10.1371/journal.pgen.1009320 (PMC7920342; doi:10.1371/journal.pgen.1009320)
Supplement: S1 Fig — Dotted lines indicate border between dental epithelium and mesenchyme. Epi, epithelium; Mes, mesenchyme. Scale bars: 100μm. (PDF) [file pgen.1009320.s001.pdf]

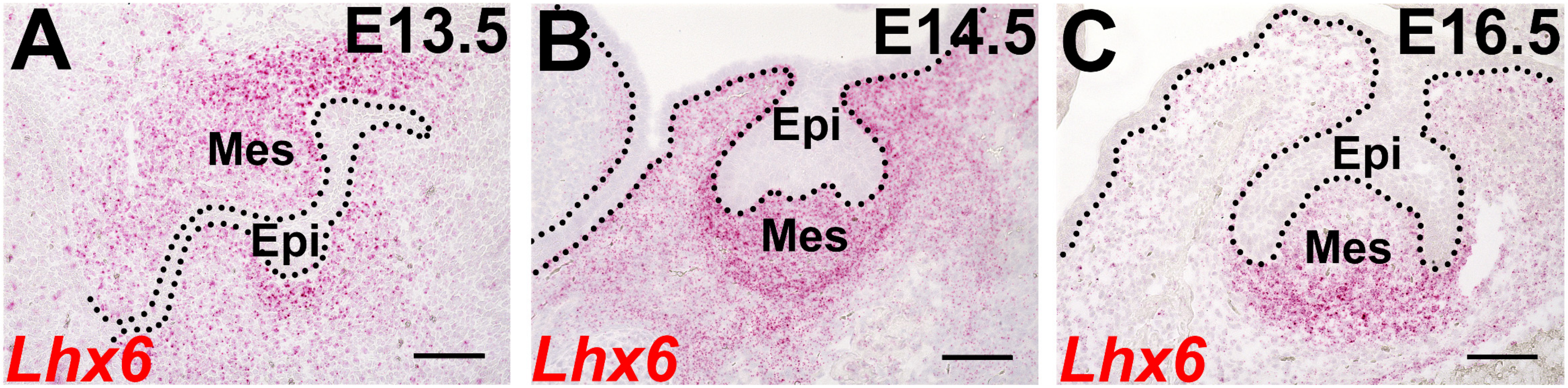

S1 Fig. Expression pattern of *Lhx6* during tooth crown development. Dotted lines indicate border between dental epithelium and mesenchyme. Epi, epithelium; Mes, mesenchyme. Scale bars: 100μm.
