# Supplementary material for: Lhx6 regulates canonical Wnt signaling to control the fate of mesenchymal progenitor cells during mouse molar root patterning
Source: PLoS Genet. 2021 Feb 17;17(2):e1009320. doi: 10.1371/journal.pgen.1009320 (PMC7920342; doi:10.1371/journal.pgen.1009320)
Supplement: S2 Fig — (A-B) Lhx6-/- mice were born alive and appeared grossly normal in the first week after birth, but developed an obvious body size reduction and failed to survive past 1 month. (C-D) Immunofluorescence staining of Lhx6 on coronal mouse molar sections. (E-F) H&E staining of mandibular first molars of control (E) and Lhx6-/- (F) mice at PN4.5 on sagittal sections. Scale bars: 100μm in C and D; 200μm in E and F. (PDF) [file pgen.1009320.s002.pdf]

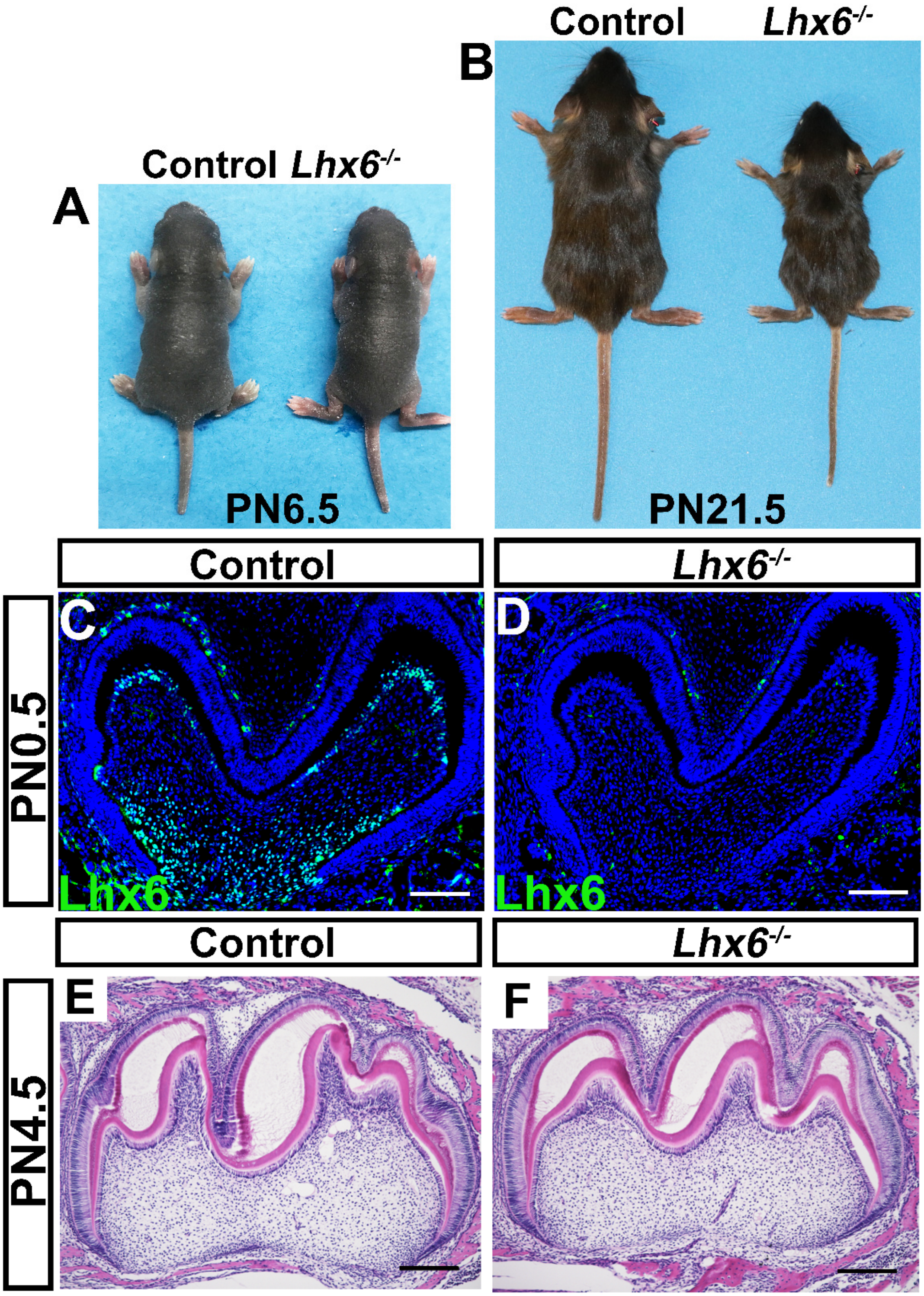

S2 Fig. *Lhx6*$^{-/-}$ mice show a reduction in body size and unaffected tooth crown morphology. (A-B) *Lhx6*$^{-/-}$ mice were born alive and appeared grossly normal in the first week after birth, but developed an obvious body size reduction and failed to survive past 1 month. (C-D) Immunofluorescence staining of Lhx6 on coronal mouse molar sections. (E-F) H&E staining of mandibular first molars of control (E) and *Lhx6*$^{-/-}$ (F) mice at PN4.5 on sagittal sections. Scale bars: 100μm in C and D; 200μm in E and F.
