# Supplementary material for: Lhx6 regulates canonical Wnt signaling to control the fate of mesenchymal progenitor cells during mouse molar root patterning
Source: PLoS Genet. 2021 Feb 17;17(2):e1009320. doi: 10.1371/journal.pgen.1009320 (PMC7920342; doi:10.1371/journal.pgen.1009320)
Supplement: S4 Fig — (A-B) TUNEL assays of control and Lhx6-/- mice at PN7.5 on coronal sections. (C-F) Immunofluorescence staining of epithelial marker Krt14 in control and Lhx6-/- mice at PN8.5 on coronal sections. Boxes in C and D are shown at higher magnification in E and F, respectively. Asterisk indicates the absence of epithelial fusion and dissociation. Scale bars: 100μm in A-D; 20μm in E-F. (PDF) [file pgen.1009320.s004.pdf]

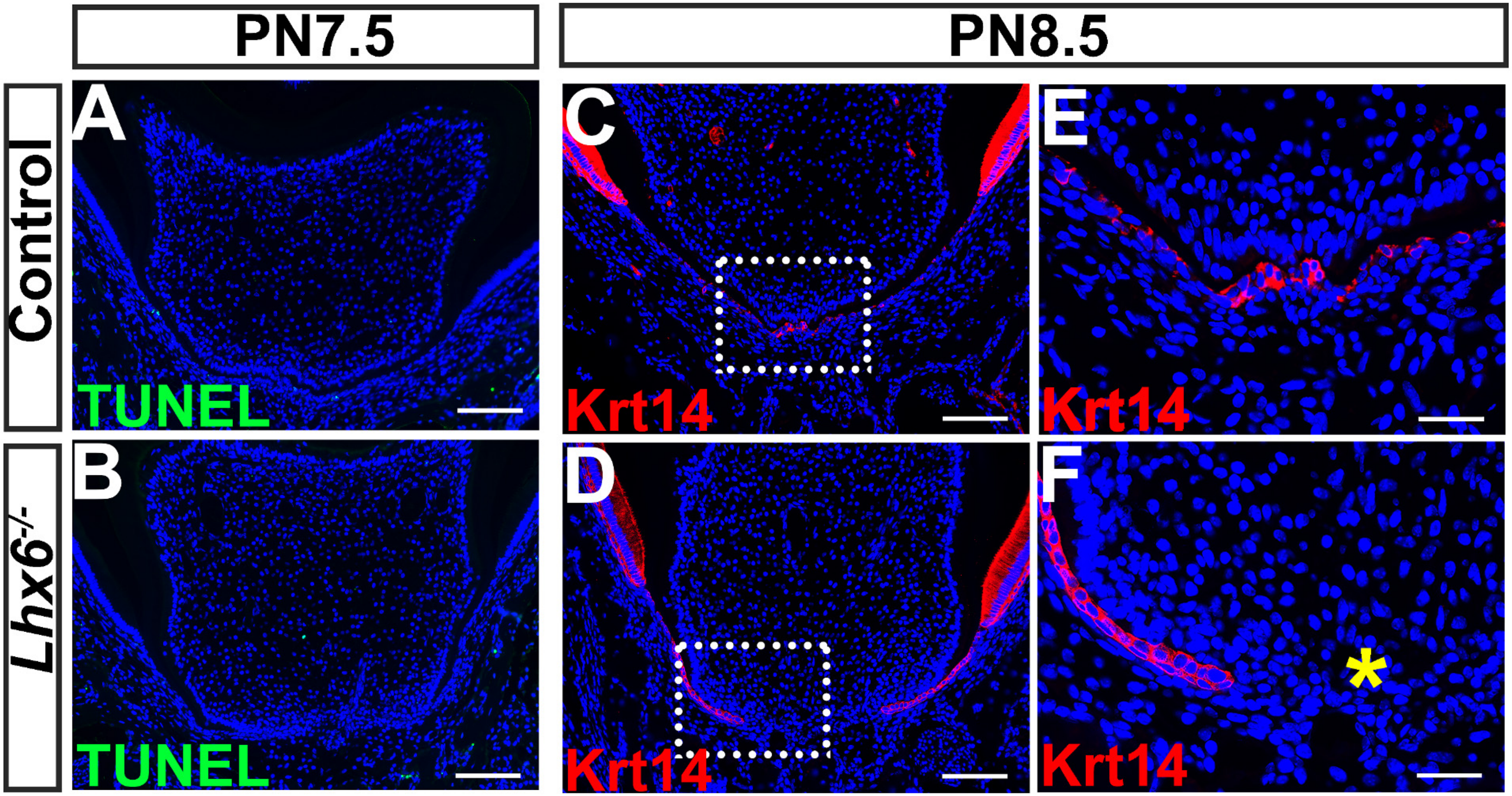

S4 Fig. Loss of *Lhx6* causes secondary epithelial defects through tissue-tissue interaction. (A-B) TUNEL assays of control and *Lhx6*$^{-/-}$ mice at PN7.5 on coronal sections. (C-F) Immunofluorescence staining of epithelial marker Krt14 in control and *Lhx6*$^{-/-}$ mice at PN8.5 on coronal sections. Boxes in C and D are shown at higher magnification in E and F, respectively. Asterisk indicates the absence of epithelial fusion and dissociation. Scale bars: 100μm in A-D; 20μm in E-F.
