# Supplementary material for: Lhx6 regulates canonical Wnt signaling to control the fate of mesenchymal progenitor cells during mouse molar root patterning
Source: PLoS Genet. 2021 Feb 17;17(2):e1009320. doi: 10.1371/journal.pgen.1009320 (PMC7920342; doi:10.1371/journal.pgen.1009320)
Supplement: S5 Fig — (A-H) X-gal staining of coronal sections of Gli1-LacZ reporter mouse molars at indicated stages. Coronal sections were analyzed. FDR, furcation development region; NFDR, non-furcation development region. Scale bars: 500μm in A, C, E, G; 50μm in B, D, F, H. (PDF) [file pgen.1009320.s005.pdf]

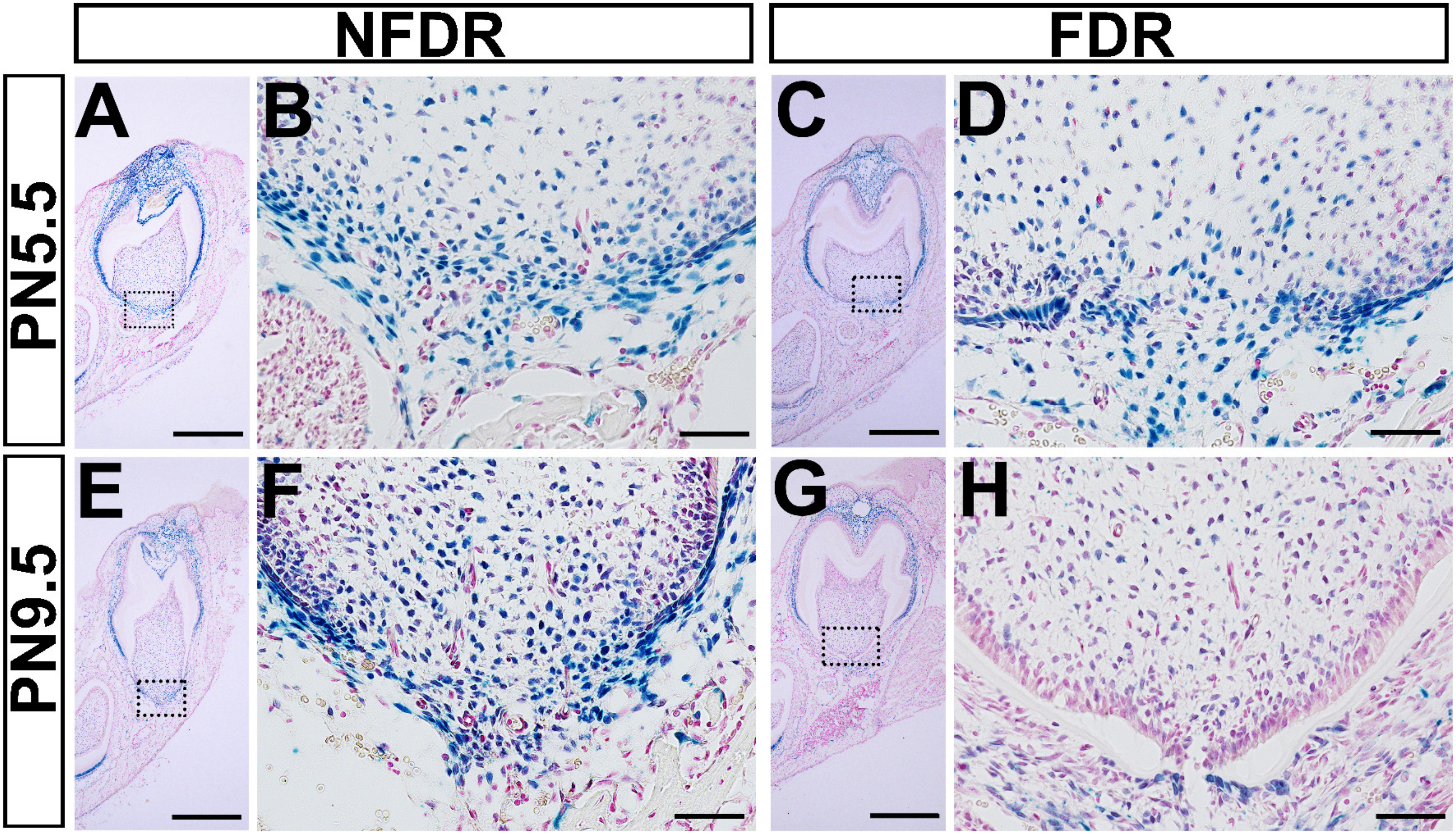

S5 Fig. Distribution patterns of Gli1+ cells between FDR and NFDR at indicated stages. (A-H) X-gal staining of coronal sections of *Gli1-LacZ* reporter mouse molars at indicated stages. Coronal sections were analyzed. FDR, furcation development region; NFDR, non-furcation development region. Scale bars: 500μm in A, C, E, G; 50μm in B, D, F, H.
