# Supplementary material for: Lhx6 regulates canonical Wnt signaling to control the fate of mesenchymal progenitor cells during mouse molar root patterning
Source: PLoS Genet. 2021 Feb 17;17(2):e1009320. doi: 10.1371/journal.pgen.1009320 (PMC7920342; doi:10.1371/journal.pgen.1009320)
Supplement: S6 Fig — (A) Heatmap hierarchical clustering showing the gene expression profiles of FDR in control and Lhx6-/- mice at PN4.5. (B) Pathway analysis of differentially expressed genes using the PANTHER tool. Only the top ten pathways in which differentially expressed genes were enriched are shown. (C-N) RNAscope assays of Smoc2 (C-F), Sfrp2 (G-J) and Frzb (K-N) of PN4.5 control mice. Coronal sections were analyzed. Boxes in C, D, G, H, K, and L are shown at higher magnification in E, F, I, J, M and N, respectively. Dotted lines in E, I, M and N indicate border between dental epithelium and mesenchyme. Arrows in E, I and M indicate regions with lower gene expression levels, while asterisks in F, J and N indicate regions with high gene expression levels. Scale bars:100μm in C, D, G, H, K and L; 20μm in E, F, I, J, M and N. FDR, furcation development region; NFDR, non-furcation development region. (PDF) [file pgen.1009320.s006.pdf]

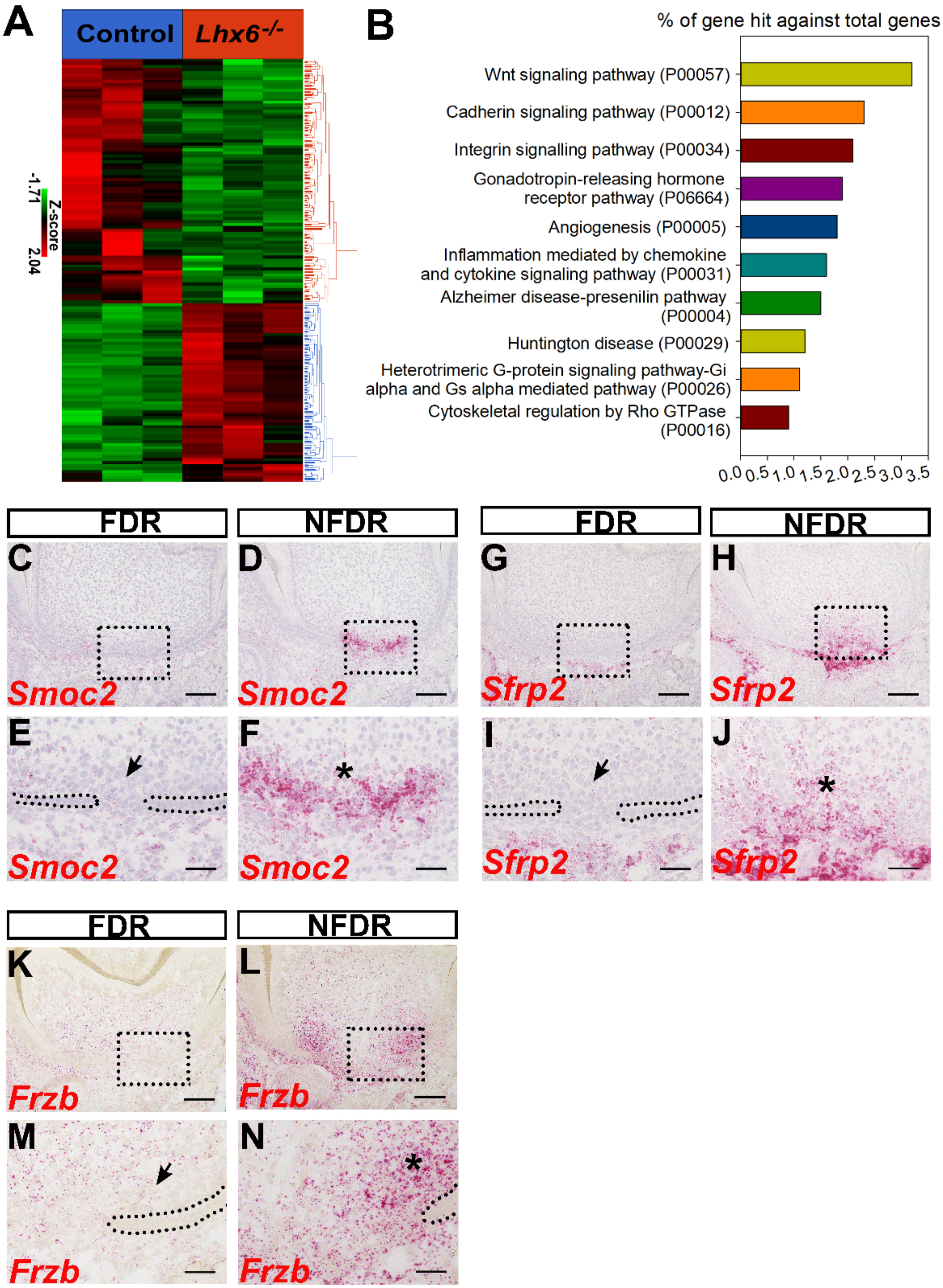

S6 Fig. Spatial expression patterns of *Smoc2*, *Sfrp2* and *Frzb* in FDR and NFDR of control mice. (A) Heatmap hierarchical clustering showing the gene expression profiles of FDR in control and *Lhx6*$^{-/-}$ mice at PN4.5. (B) Pathway analysis of differentially expressed genes using the PANTHER tool. Only the top ten pathways in which differentially expressed genes were enriched are shown. (C-N)

RNAscope assays of *Smoc2* (C-F), *Sfrp2* (G-J) and *Frzb* (K-N) of PN4.5 control mice. Coronal sections were analyzed. Boxes in C, D, G, H, K, and L are shown at higher magnification in E, F, I, J, M and N, respectively. Dotted lines in E, I, M and N indicate border between dental epithelium and mesenchyme. Arrows in E, I and M indicate regions with lower gene expression levels, while asterisks in F, J and N indicate regions with high gene expression levels. Scale bars:100μm in C, D, G, H, K and L; 20μm in E, F, I, J, M and N. FDR, furcation development region; NFDR, non-furcation development region
