# Supplementary material for: Lhx6 regulates canonical Wnt signaling to control the fate of mesenchymal progenitor cells during mouse molar root patterning
Source: PLoS Genet. 2021 Feb 17;17(2):e1009320. doi: 10.1371/journal.pgen.1009320 (PMC7920342; doi:10.1371/journal.pgen.1009320)
Supplement: S7 Fig — In situ RNAscope assays of Wnt10a (A-D), Wnt4 (E-H), Wnt6 (I-L), and Wnt3a (M-P) of control mice and Lhx6-/- mice at PN4.5. Coronal sections were analyzed. Dotted lines indicate border between dental epithelium and mesenchyme. Boxes in A, C, E, G, I, K, M and O are shown at higher magnification in B, D, F, H, J, L, N and P respectively. Scale bars: 100μm in A, C, E, G, I, K, M and O; 20μm in B, D, F, H, J, L, N and P. (PDF) [file pgen.1009320.s007.pdf]

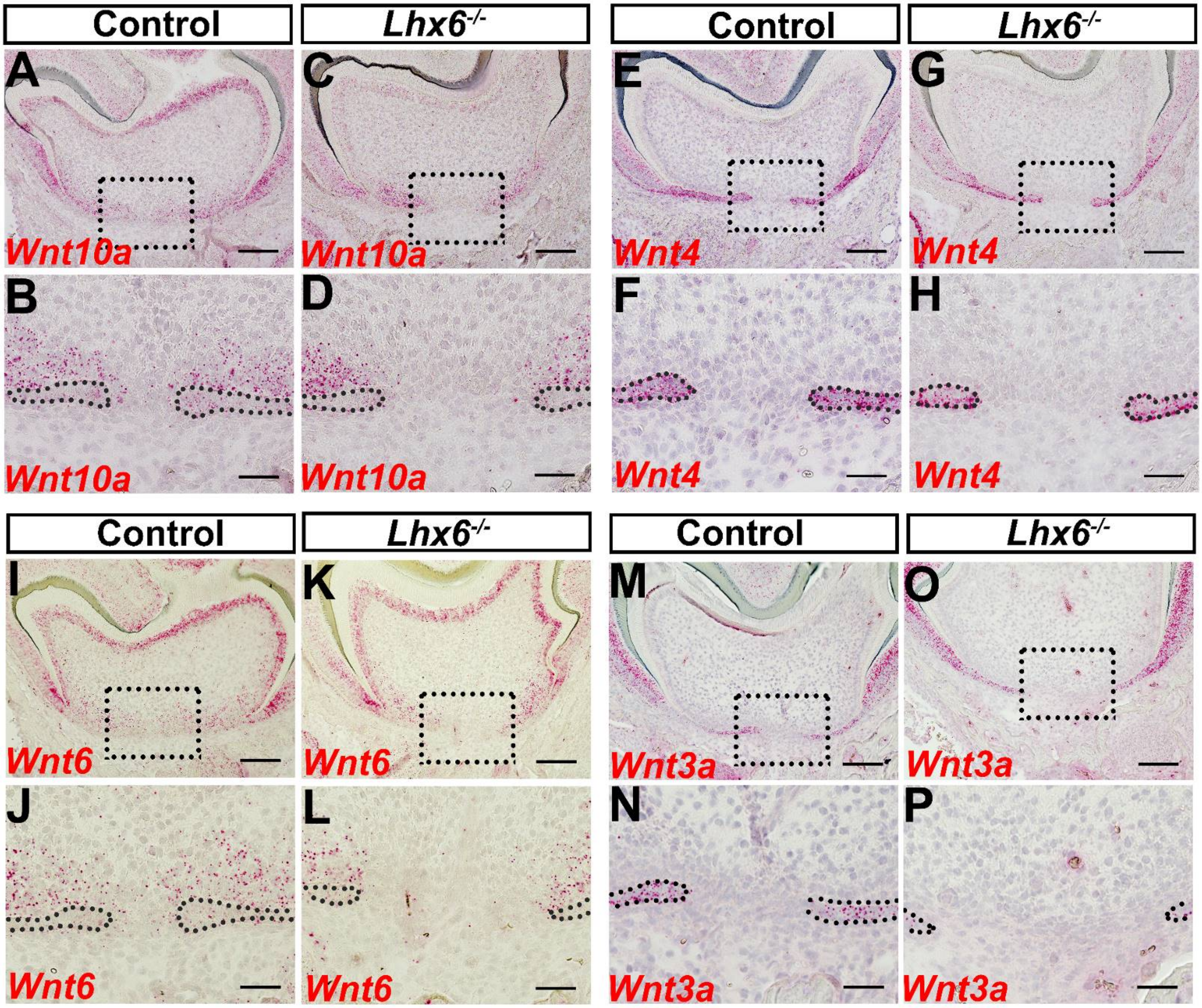

S7 Fig. No significant difference observed in the expression levels of Wnt ligands between controls and *Lhx6*$^{-/-}$ mice. *In situ* RNAscope assays of *Wnt10a* (A-D), *Wnt4* (E-H), *Wnt6* (I-L), and *Wnt3a* (M-P) of control mice and *Lhx6*$^{-/-}$ mice at PN4.5. Coronal sections were analyzed. Dotted lines indicate border between dental epithelium and mesenchyme. Boxes in A, C, E, G, I, K, M and O are shown at higher magnification in B, D, F, H, J, L, N and P respectively. Scale bars: 100μm in A, C, E, G, I, K, M and O; 20μm in B, D, F, H, J, L, N and P.
