# Supplementary material for: Lhx6 regulates canonical Wnt signaling to control the fate of mesenchymal progenitor cells during mouse molar root patterning
Source: PLoS Genet. 2021 Feb 17;17(2):e1009320. doi: 10.1371/journal.pgen.1009320 (PMC7920342; doi:10.1371/journal.pgen.1009320)
Supplement: S8 Fig — microCT scanning of maxillary and mandibular first molars of PN21.5 control (A-D) and Lhx6-CreER/+;Ctnnb1floxE3/+ (E-H) mice. Scale bars: 200μm in A, C, E, G; 100μm in B, F; 80μm in D and H. The schematic at the bottom indicates the induction protocol. TMX: tamoxifen. (PDF) [file pgen.1009320.s008.pdf]

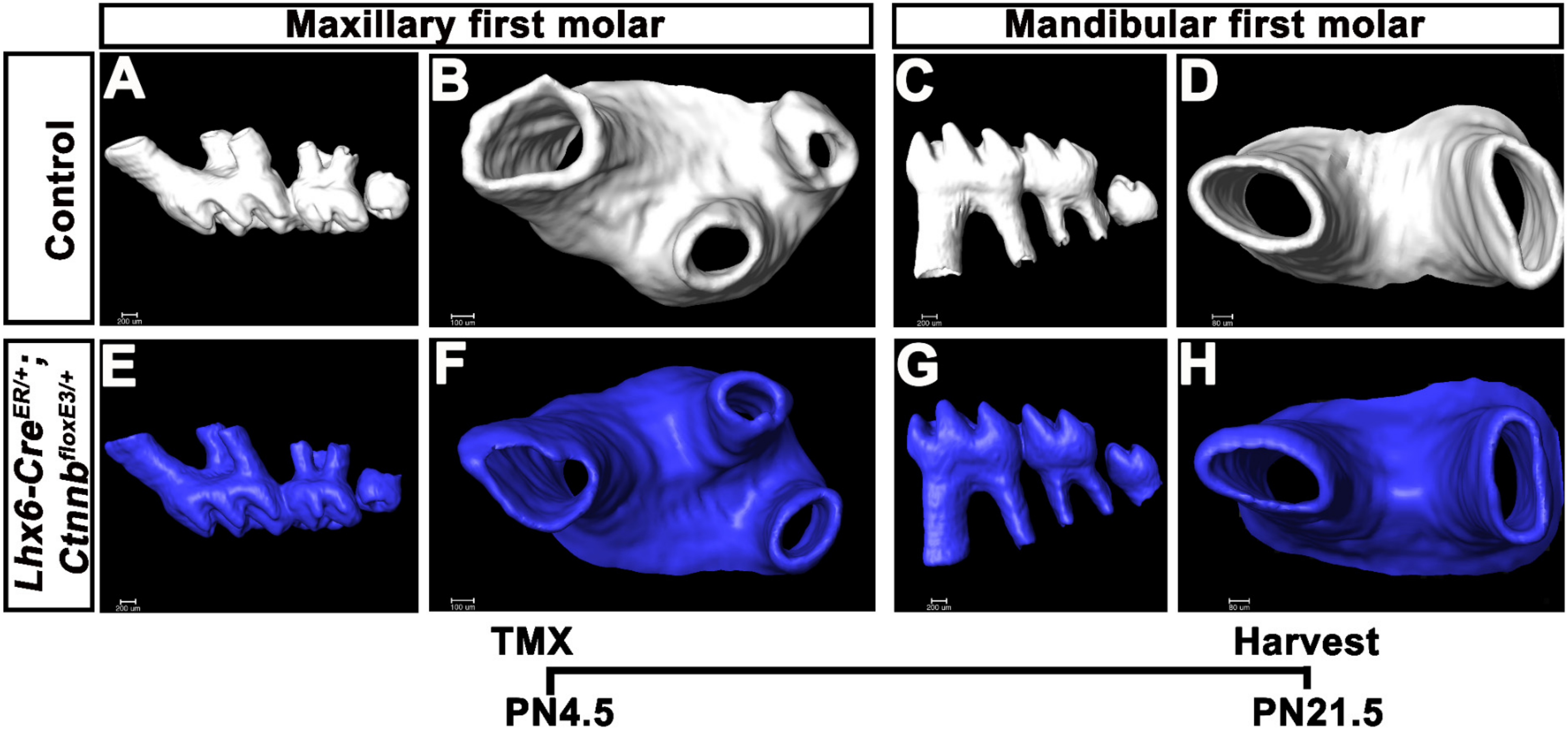

S8 Fig. Compound heterozygous mice have no abnormal furcation phenotype. microCT scanning of maxillary and mandibular first molars of PN21.5 control (A-D) and *Lhx6-Cre*$^{ER/+;}$*Ctnnb1*$^{floxE3/+}$ (E-H) mice. Scale bars: 200μm in A, C, E, G; 100μm in B, F; 80μm in D and H. The schematic at the bottom indicates the induction protocol. TMX: tamoxifen.
