# Supplementary material for: Lhx6 regulates canonical Wnt signaling to control the fate of mesenchymal progenitor cells during mouse molar root patterning
Source: PLoS Genet. 2021 Feb 17;17(2):e1009320. doi: 10.1371/journal.pgen.1009320 (PMC7920342; doi:10.1371/journal.pgen.1009320)
Supplement: S9 Fig — No Lhx6 binding motif was detected in the proximal regulatory region (<50Kb) upstream or downstream of the Sfrp2 gene locus. However, there are some potential Lhx6 binding sites in the distal region as indicated by the arrows. (PDF) [file pgen.1009320.s009.pdf]

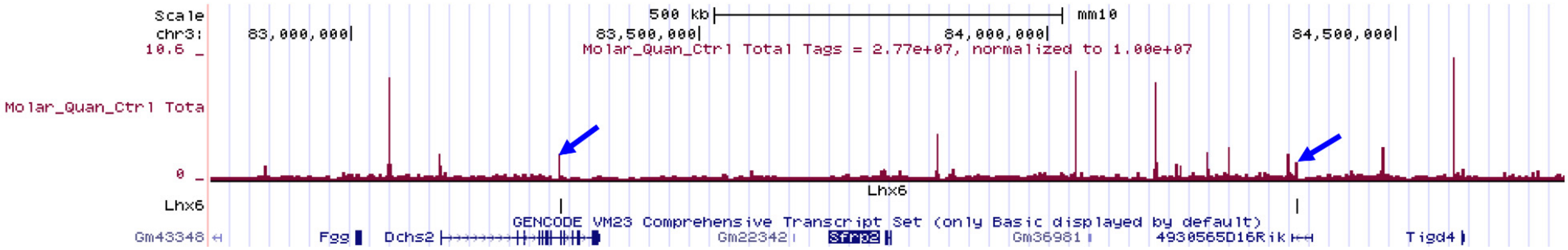

S9 Fig. ATAC-seq analysis of molars from PN7.5 wild type mice. No Lhx6 binding motif was detected in the proximal regulatory region (<50Kb) upstream or downstream of the *Sfrp2* gene locus. However, there are some potential Lhx6 binding sites in the distal region as indicated by the arrows.
