# Supplementary material for: Lhx6 regulates canonical Wnt signaling to control the fate of mesenchymal progenitor cells during mouse molar root patterning
Source: PLoS Genet. 2021 Feb 17;17(2):e1009320. doi: 10.1371/journal.pgen.1009320 (PMC7920342; doi:10.1371/journal.pgen.1009320)
Supplement: S1 Table — (PDF) [file pgen.1009320.s010.pdf]

S1 Table. Top 15 differentially expressed genes discovered via RNA-sequencing

Gene ID	Total counts	p-value	Fold change
Smoc2	8.82E+02	2.43E-12	4.9
AY036118	8.37E+01	9.81E-08	-3.2
Dclk1	1.12E+02	1.59E-07	2.0
Lypd1	5.51E+01	2.52E-07	3.5
Dio3	2.23E+02	2.67E-07	2.5
Krt18	4.20E+01	7.44E-07	2.7
Wfikkn2	1.20E+02	9.45E-07	2.4
Ntrk3	1.27E+02	1.06E-06	2.9
Nbl1	7.17E+02	1.57E-06	1.7
Frzb	2.53E+02	1.60E-06	1.6
Osr2	1.35E+02	1.63E-06	1.9
Igfbp3	2.37E+03	1.99E-06	1.9
Matn4	4.96E+02	2.31E-06	1.7
Sfrp2	9.87E+02	3.23E-06	2.1
Bmp2	1.65E+02	4.21E-06	1.7
