# Supplementary material for: Lhx6 regulates canonical Wnt signaling to control the fate of mesenchymal progenitor cells during mouse molar root patterning
Source: PLoS Genet. 2021 Feb 17;17(2):e1009320. doi: 10.1371/journal.pgen.1009320 (PMC7920342; doi:10.1371/journal.pgen.1009320)
Supplement: S2 Table — (PDF) [file pgen.1009320.s011.pdf]

S2 Table. Primary antibody information

	Antibody	Source	Cat No.	Dilution
Immuno-staining	Chicken polyclonal anti-beta Galactosidase	Abcam	ab9361	1:100
	Rabbit monoclonal anti-Cytokeratin 14	Abcam	ab181595	1:100
	Rabbit monoclonal anti-Ki67	Abcam	ab16667	1:100
	Rabbit polyclonal anti-Periostin	Abcam	ab215199	1:500
	Mouse monoclonal anti-Lhx6	Santa Cruz	Sc-271433	1:100
	anti-Rabbit Alexa Fluor 568	Life Technologies	A-11011	1:200
	anti-chicken Alexa Fluor 488	Life Technologies	A-11039	1:200
Western blot	Mouse monoclonal to turboGFP	OriGene	TA150041	1:2000
	Monoclonal ANTI-FLAG M2 antibody	Sigma	F1804	1:2000
