# Supplementary material for: Lhx6 regulates canonical Wnt signaling to control the fate of mesenchymal progenitor cells during mouse molar root patterning
Source: PLoS Genet. 2021 Feb 17;17(2):e1009320. doi: 10.1371/journal.pgen.1009320 (PMC7920342; doi:10.1371/journal.pgen.1009320)
Supplement: S3 Table — (PDF) [file pgen.1009320.s012.pdf]

S3 Table. Probe information

Probe	Source	Cat No.
RNAscope Probe-Mm-*Lhx6*	Advanced Cell Diagnostics	422791
RNAscope Probe-Mm-*Lhx8*	Advanced Cell Diagnostics	515101-C2
RNAscope Probe-Mm-*Dspp*	Advanced Cell Diagnostics	448301
RNAscope Probe-Mm-*Sfrp2*	Advanced Cell Diagnostics	400381
RNAscope Probe-Mm-*Frzb*	Advanced Cell Diagnostics	404861
RNAscope Probe-Mm-*Smoc2*	Advanced Cell Diagnostics	318541
RNAscope Probe-Mm-*Wnt10a*	Advanced Cell Diagnostics	401061
RNAscope Probe-Mm-*Wnt4*	Advanced Cell Diagnostics	401101
RNAscope Probe-Mm-*Wnt6*	Advanced Cell Diagnostics	401111
RNAscope Probe-Mm-*Wnt3a*	Advanced Cell Diagnostics	405041
RNAscope Probe-Mm-*Axin2*	Advanced Cell Diagnostics	400331
