# Supplementary material for: Lhx6 regulates canonical Wnt signaling to control the fate of mesenchymal progenitor cells during mouse molar root patterning
Source: PLoS Genet. 2021 Feb 17;17(2):e1009320. doi: 10.1371/journal.pgen.1009320 (PMC7920342; doi:10.1371/journal.pgen.1009320)
Supplement: S4 Table — (PDF) [file pgen.1009320.s013.pdf]

S4 Table. Primer information

Gene		Primer sequence
Gapdh	Forward	TGGATTTGGACGCATTGGTC
	Reverse	TTTGCACTGGTACGTGTTGAT
Cdkn1c	Forward	CGAGGAGCAGGACGAGAATC
	Reverse	GAAGAAGTCGTTCGCATTGGC
Axin2	Forward	TGACTCTCCTTCCAGATCCCA
	Reverse	TGCCCACACTAGGCTGACA
Dspp	Forward	ATTCCGGTTCCCCAGTTAGTA
	Reverse	CTGTTGCTAGTGGTGCTGTT
Smoc2	Forward	CCCAAGCTCCCCTCAGAAG
	Reverse	GCCACACACCTGGACACAT
Sfrp2	Forward	CGTGGGCTCTTCCTCTTCG
	Reverse	ATGTTCTGGTACTCGATGCCG
Frzb	Forward	CACAGCACCCAGGCTAACG
	Reverse	TGCGTACATTGCACAGAGGAA
